# Ultra High Content Image Analysis and Phenotype Profiling of 3D Cultured Micro-Tissues

**DOI:** 10.1371/journal.pone.0109688

**Published:** 2014-10-07

**Authors:** Zi Di, Maarten J. D. Klop, Vasiliki-Maria Rogkoti, Sylvia E. Le Dévédec, Bob van de Water, Fons J. Verbeek, Leo S. Price, John H. N. Meerman

**Affiliations:** 1 Division of Toxicology, Leiden Academic Center for Drug Research, Leiden University, Leiden, The Netherlands; 2 OcellO B.V., Bio Partner Center Leiden, Leiden, The Netherlands; 3 Imaging and BioInformatics, Leiden Institute of Advanced Computer Science, Leiden University, Leiden, The Netherlands; Sanford Burnham Medical Research Institute, United States of America

## Abstract

In many situations, 3D cell cultures mimic the natural organization of tissues more closely than 2D cultures. Conventional methods for phenotyping such 3D cultures use either single or multiple simple parameters based on morphology and fluorescence staining intensity. However, due to their simplicity many details are not taken into account which limits system-level study of phenotype characteristics. Here, we have developed a new image analysis platform to automatically profile 3D cell phenotypes with 598 parameters including morphology, topology, and texture parameters such as wavelet and image moments. As proof of concept, we analyzed mouse breast cancer cells (4T1 cells) in a 384-well plate format following exposure to a diverse set of compounds at different concentrations. The result showed concentration dependent phenotypic trajectories for different biologically active compounds that could be used to classify compounds based on their biological target. To demonstrate the wider applicability of our method, we analyzed the phenotypes of a collection of 44 human breast cancer cell lines cultured in 3D and showed that our method correctly distinguished basal-A, basal-B, luminal and ERBB2+ cell lines in a supervised nearest neighbor classification method.

## Introduction

Over the past decade, *in vivo* models and 2D cell cultures represented the two principle approaches used to study cellular processes. The extreme low throughputs of *in vivo* models and poor (patho-) physiological relevance of over-simplified monolayer cell cultures motivated the development of 3D cell culture methods. In many situations, 3D cell cultures mimic the natural organization of tissues more closely than 2D cultures, enabling cells to develop complex micro-tissue phenotypes. Especially for the study of tissue development where the spatial organization, architecture and interaction with the extracellular matrix are critical, 3D cell culture models may bridge the gap between *in vivo* studies and simple 2D cell mono-layer cultures [1], [2], 3D cell cultures are also used frequently in tumor studies, allowing the effects of ECM, stromal cells and individual genes on tumor growth and invasion to be studied [3]–[7].

One potential application of 3D cultures is for the high-throughput screening (HTS) and high content analysis (HCA) of pharmacologically active compounds [8]. For such purposes, 3D cultures are treated with compound libraries in 96- or 384-wells micro plates. Here, 3D cultures have presented a challenge in collecting image data with sufficient resolution because acquiring high resolution images is time consuming and therefore not always feasible for HTS. Many methodologies have been established for the image analysis of HTS [9], although the quantification of phenotypes is mostly performed with single or multiple simple parameters. This is not sufficient to study of the full range of effects of test compounds.

Our goal was to develop an automated multi-parametric profiling platform which is suited for HTS and is able to quantify cellular phenotypes exhaustively. Such a platform should apply rapid image preprocessing and segmentation methods that are suited for images with limited resolution. More importantly, this platform should be able to recover heterogeneous cell behavior. For example, it should be able to distinguish epithelial cells that develop branched structures from those that do not in response to a specific treatment. Such cell-to-cell heterogeneity seems essential for the plasticity of tissue responses e.g. in response to inflammation and associated with tumor invasion [10], [11].

**Figure 1:**
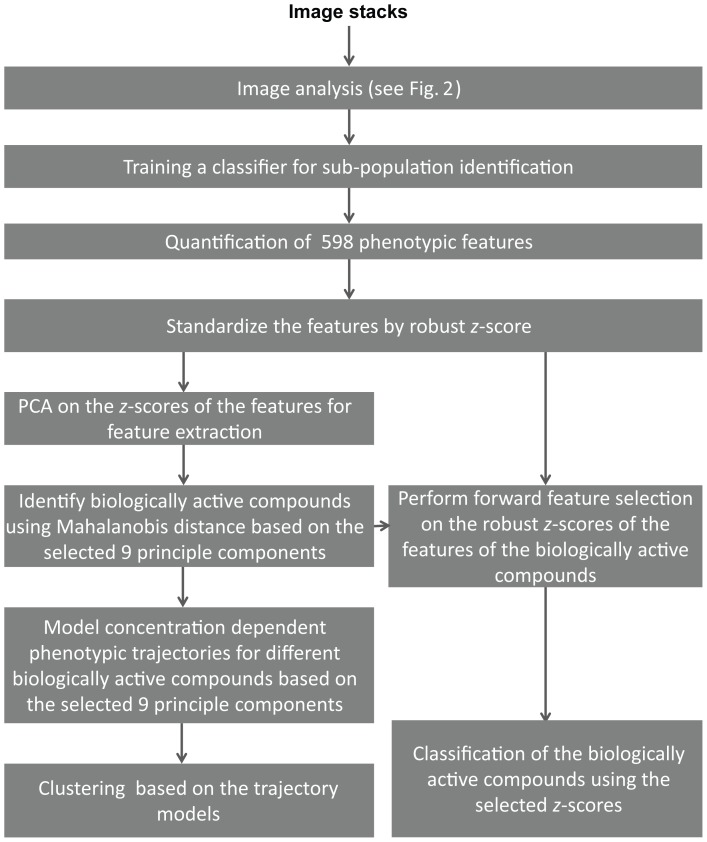
An overview of the project workflow. The details are explained in the Methods and Results of the manuscript and more information on the individual data analysis steps can be found in the File S1.

As proof of concept, we investigated mouse breast cancer cells (4T1) after they formed micro-tissues in 3D by monitoring their cellular phenotypic response to a diverse set of compounds, using a novel 3D screening and ultra high content analysis (uHCA) technique. An overview of the project workflow is presented in the Figure 1. After image acquisition, we extensively mined images for feature data which we used for multi-parametric phenotype profiling. To investigate phenotypic patterns, principle component analysis (PCA) was first used to reduce the dimensionality of the dataset. Subsequently, we compared various multi-parametric tests to identify biologically active compounds. Next, polynomial regression modeling was applied to characterize concentration dependent trajectories for each biologically active compound, and the distance between the trajectories was used for hierarchical clustering of compounds. Finally, multiple classification models were used to identify distinct phenotypic patterns.

## Methods

An overview of the project workflow is presented in Figure 1 and more information on the individual data analysis steps can be found in File S1.

### Cell culturing, fluorescence staining, and image acquisition

To generate 3D micro-tissues, mouse triple negative breast cancer cells (4T1) were obtained from the American Type Culture Collection (ATCC) (Manassas, VA, USA) and were cultured in a mixture of collagen type IV and laminin-rich basement membrane extract (Matrigel) for 4 days in 384-well high content imaging micro plates. 24 hours after seeding, cells were exposed to 29 different compounds with different biological activities (Table S1) at 6 different concentrations (0.03 µM, 0.1 µM, 0.316 µM, 1 µM, 3.16 µM, 10 µM) in quadruplicate (See Figure S1 for plate layout). Replicates were located on the same plates. Cells growing in 0.2% DMSO without any compound treatment were included as controls. There were 24 control wells in each of the 384-well micro plates. To avoid an edge effect, the first and last two rows and columns were left empty.

After 72 hours of exposure, the cultured micro-tissues were fixed and stained with Hoechst 33258 (final concentration 0.4 µg/mL) and Rhodamine-phalloidin (final concentration 0.1 µM) to visualize nuclei and F-actin, respectively.

For the classification of the 44 breast cancer cell lines, these were cultured and stained (at 96 hr) similarly as described above for the 4T1 cells. The human breast cancer cell lines were from ATCC or as described [12] and provided to us by Prof. Dr. John A. Foekens and Dr. John W. Martens from Erasmus University Medical Center-Daniel den Hoed Cancer Center, Rotterdam, The Netherlands.

For each well of a 384-well plate, 2 channels (corresponding to Rhodamine and Hoechst fluorescence) of 16-bit image stacks were collected using an automated microscope system: BD pathway 855, equipped with a 4X magnification/0.16NA UPlanSApo objective. For each image slice (n = 17), pixel size was 1.6 µm and step size in z direction was 50 µm

### 2D projection

**Figure 2:**
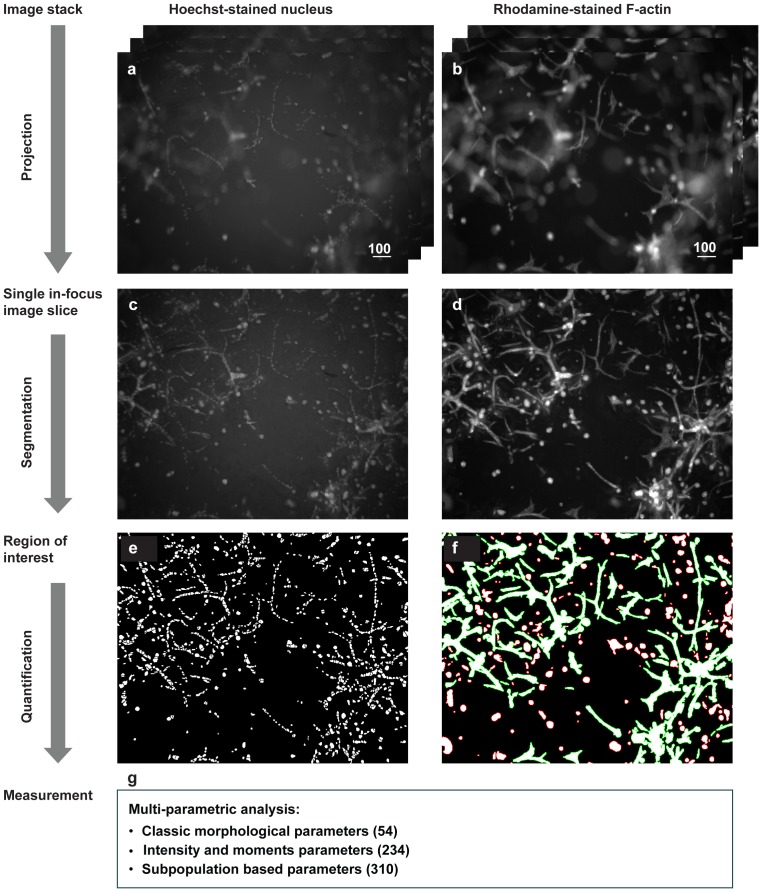
Stepwise demonstration of the image analysis method. Scale bar represents 100 micrometer. (a) Image stack obtained from the Hoechst stained nuclei channel. (b) Image stack obtained from the Rhodamine stained F-actin channel. (c) In-focus 2D image projected from the stacks of Hoechst stained nuclei channel. (d) In-focus 2D image projected from the stacks of Rhodamine stained F-actin channel. (e) Binary nuclear mask after segmentation by Watershed Masked Clustering. (f) Binary cellular mask after segmentation. The subpopulation classification result is also shown here. The green contour represents branched and interconnected complex networks. The red contour represents spherical colonies. (g) Quantitative parameters measured for each well of the 384-well plates. See Methods for further description.

In order to achieve high-throughput in our methodology, we used wide-field microscopy for screening as its imaging process is much faster than confocal laser scanning microscopy. However, due to its limited depth of field, each image slice obtained from a wide-field microscope includes both in-focus regions and out-of-focus regions of micro-tissue specimen (Figure 2a, 2b) that are bigger than the depth of field. To extract only the in-focus information, a free open source plugin of ImageJ “Stack Focuser” was used to compose 2D image slices (Figure 2c, 2d) by projecting only in-focus regions from each slice of image stacks (details see File S1).

### Segmentation

For the Hoechst stained nuclei channel, our novel Watershed Masked Clustering [13], [14] was applied to retrieve the binary masks for individual nuclear regions. This algorithm first generates watersheds on the Gaussian filter (σ = 2.0) convolved images to separate the adjacent nuclei into individual compartments. Convolving with a Gaussian filter prevented the influence of noise from causing artificial local maxima. Next, K-means clustering was applied on the images prior to convolving to refine the region of the nucleus in each compartment (Figure 2e, File S1).

For the Rhodamine stained F-actin channel, median filter and rolling ball were applied before segmentation to remove the background and reduce the noise level. The radius of rolling ball was chosen to be slightly bigger than the smallest cell colonies. Next, the local Niblack algorithm [15] was used to define regions of cell colonies (Figure 2f, File S1).

### Multi-parametric profiling

Quantification algorithms assembled from the literature [16], [17] were incorporated into an ImageJ plugin to extract different morphological and fluorescence intensity parameters from the images (Figure 2g, Table S2). We observed that the 3D micro-tissues formed different subpopulations: 1) spherical colonies and 2) branched and interconnected complex network. Upon exposure to different compounds, the proportion and the shape of these two subpopulations often changed in a specific pattern. To quantitatively study these changes, an automated classifier for these two subpopulations was developed and embedded in the image analysis pipeline (see File S1, Figure S2). Relevant information was collected for each subpopulation based on fluorescence intensity and morphology (Table S2). In total, 598 parameters were measured from the images of each well for the whole population and the two subpopulations. A complete description can be found in File S1.

Software and all image data are available at: http://dx.doi.org/10.4121/uuid:d5b91e46-07e7-4077-bd63-3fa2b82c847f

## Results

### 4T1 breast cancer cells acquire a complex phenotype in 3D culture, which is perturbed by biologically active compounds

Images obtained from the control wells showed that the 4T1 cells spontaneously formed a heterogeneous array of multi-cellular structures comprising spheroids and branched micro-tissues that were interconnected to form a complex network (Figure S3a). Cells were exposed to 29 test compounds including tyrosine kinase inhibitors, cytostatic drugs, and Wnt-signalling activators (Table S1). Exposure to many of the 29 test compounds resulted in a change in various aspects of network formation after 72 hours, such as branch length and thickness, number of branches, and the proportion and shape of spheroids (Figure S3b–f). Some compounds, such as the protease inhibitor bortezomib, and several compounds at higher concentrations, showed apparent toxicity, characterized by complete inhibition of network formation and pronounced inhibition of cell growth (Figure S3f).

### Identification of biologically active compounds

The primary goal of our present HTS method was to identify biologically active compounds which significantly affect the cellular phenotype compared to controls. To remove between-plate variation, we firstly performed cross-plate normalization by calculating the robust *z*-score [18] for each of 598 measured parameters (see File S1). The normalized *z*-scores were used for principle component analysis (PCA) and 9 principle components were obtained to preserve 90% data variation. A 3D plot of data points on the first 3 principle components is given in Figure S4. The parameters that contribute most to the first 3 principle components are shown in Table S3. These include subpopulation parameters, intensity parameters and morphological parameters. To identify biologically active compounds, we compared three multi-parametric tests based on the 9 principle components: Mahalanobis distance, Chi-square and Wilks' lambda test (see File S1). Mahalanobis distance at α = 0.05 came the closest to visual scoring a treatment as having an effect on phenotype. Figure S5a shows the false positives and false negatives for the different statistical tests. The Mahalanobis distance to control (DMSO) of all active compounds is shown in Figure S5b. Table S4 lists the active compounds (21 out of 29) and the concentrations at which a statistically significant effect on the phenotype was detected.

### Concentration dependent phenotypic trajectories of biologically active compounds

**Figure 3:**
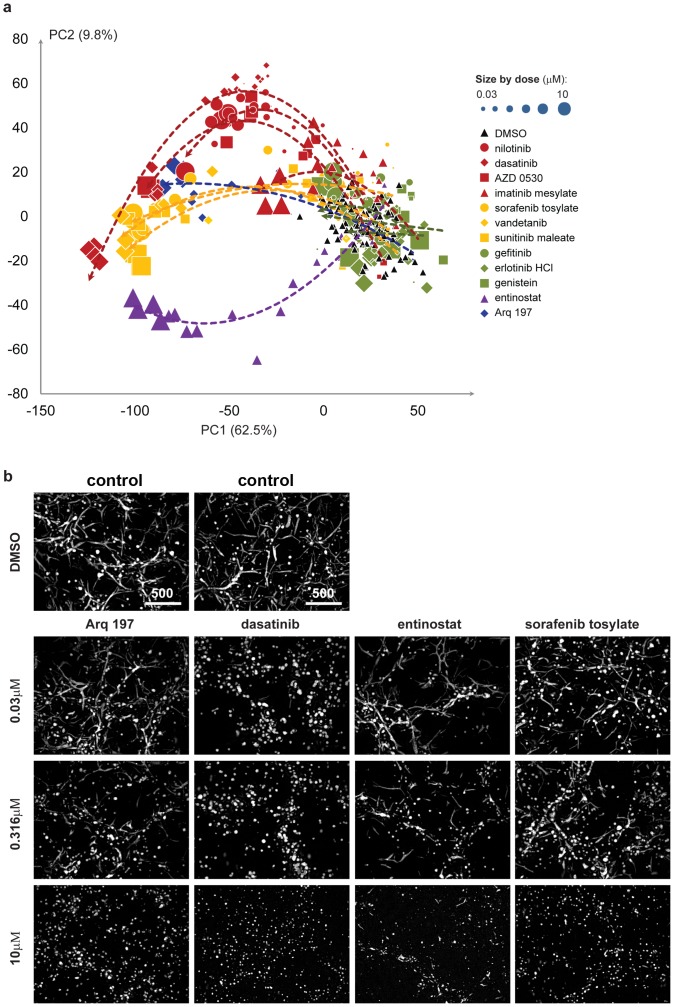
2D PCA plot of phenotype profiles for various active compounds and their concentration dependent phenotypic trajectories. (a) 2D PCA plot of phenotype profiles for negative control (DMSO) and 12 active compounds at different concentrations. Percentages of data variation preserved in each principle component are shown with each axis. Compounds with the same biological target are colored identically. Red: BCR-ABL target inhibitor; Yellow: VEFGR inhibitor; Green: EGFR inhibitor; Purple: HDAC inhibitor; Blue: c-MET inhibitor. Concentration is represented by the size of data points. The trend lines were added for each effective compound using 2nd polynomial regression models. (b) Comparison of microscope images of four example compounds with two DMSO control images. Each compound has a different biological target. 2D projected images from the Rhoadamine stained F-actin channel are shown here. Scale bar represents 500 micrometer

We found for many biologically active compounds, that data points seem to move away from negative controls in a trajectory with increasing concentration. This is shown clearly in a PCA plot of the first 2 principle components (Figure 3a). Interestingly, the trajectories of the different active compounds separate the most at medium concentrations but converge at higher concentrations. This may be explained by the fact that at high concentrations (10 µM) severe toxicity is induced which inhibits not only cell invasion and branching but also proliferation and growth, leading to a similar phenotype. This is shown for the compounds Arq 197, dasatinib, entinostat, and sorafenib tosylate at the concentration of 10 µM (Figure 3b). Most compounds had only a marginal effect on cellular network formation at the lowest concentration that was tested (0.03 µM), except for dasatinib that already induced apparent inhibition of network formation at this concentration. At the concentration of 0.316 µM, all compounds induced distinct phenotypes. Dasatinib inhibited branch-ing but not proliferation so that bigger cell colonies were formed, while entinostat induced much thinner branches compared to control (Figure 3b). Sorafenib tosylate and Arq 197 caused formation of much shorter branches, indicative of inhibition of invasion. Most strikingly, we found that trajectories of compounds that inhibit the same biological target were more similar to each other than to trajectories of compounds with a different target (Figure 3a), indicating that phenotypic development of 4T1 cells is effected by different biological targets in characteristic ways and that we can identify this with our uHCA methodology.

### Trajectory modeling and phenotypic pattern recognition

**Figure 4:**
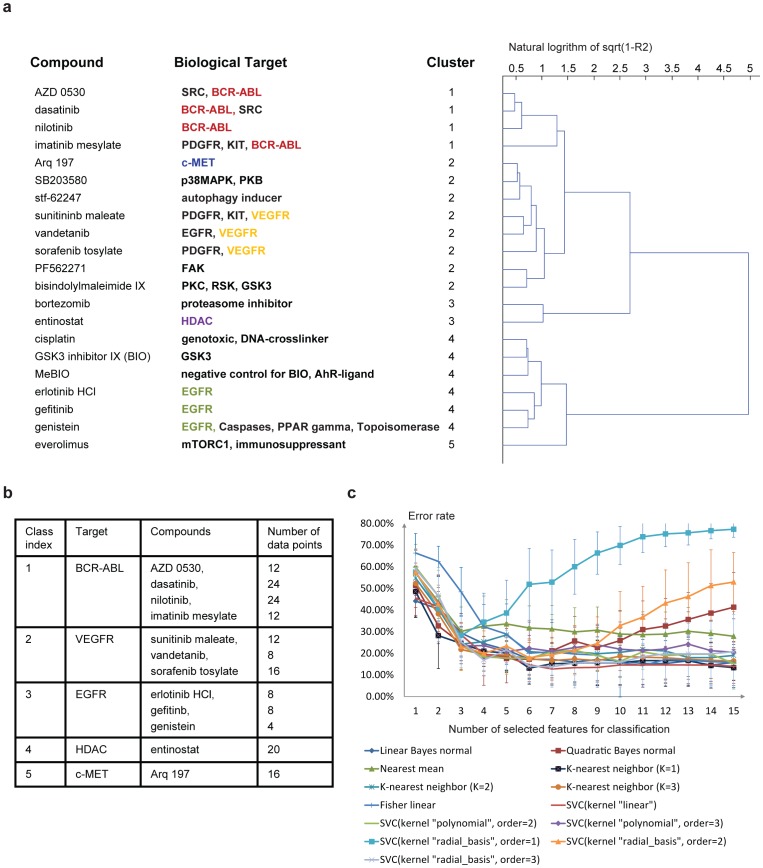
Characterization of cellular phenotype by clustering and classification. (a) Hierarchical clustering result using an average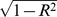 matrix as distance matrix. The scale of dendrogram is the natural logarithm of 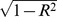. (b) Five defined classes of test compounds and corresponding compounds and number of data points. (c) Classification result using multiple classification methods. Feature selection with search algorithm “forward” and criterion “Mahalanobis distance” was applied to detect optimal number of features. For each classification method and each number of selected features, 10 fold cross-validation was repeated 10 times, resulting in 10 error rates. The average error rates are shown in the chart with standard deviation as error bar. SVC means support vector machine classification.

To further characterize the different phenotypes, we used 2nd order polynomial regression modeling to build the trajectory for each identified active compound. First, we investigated data variation for each of the 9 principle components. We compared the data between control and active compounds using a two sample Kolmogorov-Smirnov (KS) test. The principle components with no significant difference, equal data variation, or bigger variation in negative controls than in active compounds, were excluded to avoid overtraining, resulting in retaining only the first 2 components. Next, a 2D polynomial regression model of the trajectory for each compound was trained and the difference between the trajectories of two compounds 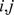 was computed based on the coefficient of determination 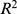 (see File S1). Subsequently, a hierarchical clustering with complete linkage [19] was applied on the distance matrix defined in the File S1 (Figure 4a). Consistent with our finding in the PCA analysis, compounds with the same biological target cluster together but compounds with different targets are separated. To further validate our hypothesis that phenotypic responses are specific to the biological target that is inhibited, we applied classification. Five classes of compounds were defined based on their biological target (Figure 4b). Only active concentrations were taken into account, and compound classes with less than 15 data points were not included in order to avoid the curse of dimensionality. We tested several classification algorithms on the 598 z-score space, including k-nearest neighbor classification, linear Bayes normal classification [21], [21,22] quadratic Bayes normal classification [20], [22] nearest mean classification, support vector machine classification (SVC) [23], [24] with different kernels, and Fisher linear classification [20], [22], [25]. Before classification, a forward feature selection with the Mahalanobis distance metric was performed and only the selected features were used for classification. To evaluate the performance of feature selection and classification, 10-fold cross validation was used. For each of 10 tests, a classification error rate (%) was calculated as follows: 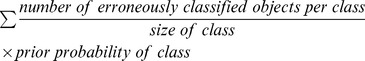
(1)where the prior probability is equal for each class (20%). According to the feature selection result, we found that the most frequently selected discriminative parameters included morphology- and intensity-based parameters and parameters from both the whole population and the two subpopulations. The classification result is shown in Figure 4c.

The lowest mean classification error rate of 12.8% was obtain from a linear kernel based SVC with 7 features selected. 1-nearest neighbor classification also showed a relatively high classification accuracy with an error rate of 13.1% when 6 features were selected. With this high classification accuracy of different classification methods, further evidence is provided that our method may be used to identify the biological targets of compounds.

### Contribution of the different parameter classes to the classification

To recover the heterogeneity of all responses of the 4T1 cells to the different compounds, we had analyzed different multi-cellular subpopulations and quantified phenotype parameters for each subpopulation separately. In order to establish the value of analyzing these subpopulations separately, we repeated the above analysis excluding subpopulation-related parameters, collecting only 288 parameters from each well. The result showed that excluding subpopulation parameters caused a failure to co-cluster all BCR-ABL inhibitors together, and EGFR inhibitors together. We also repeated classification without subpopulation parameters and the classification accuracy was decreased; SVC with linear kernel still performed relatively better than other classification methods when 8 features were selected, but the error rate increased to 16.5% compared to the error rate of 12.8% when subpopulation parameters were used.

We also repeated the analysis without moments and intensity parameters. Only morphological parameters were measured from the whole object population and the two subpopulations, resulting in 152 parameters extracted from each well for classification. The classification results of SVC with linear kernel showed that omission of moments and intensity parameters increased the misclassification error to 15.4%. These results show that simplification of the analysis by omitting either subpopulation parameters or intensity and moments parameters e.g. to decrease time for computation, compromises the quality of the analysis.

### Comparison to other analysis methods

In order to compare the performance of our newly developed analysis method with other published methods, we analyzed our images with PhenoRipper [26] and CellProfiler [27]. PhenoRipper is a platform using a segmentation-free approach: it breaks down images into small blocks and clusters the blocks to different types according to the pixel intensity distribution. Then it quantifies images by proportions of different types of block. As the quantification is not executed based on segmentation, this approach is highly computational efficient, using ∼5 minutes to analyze four complete 384 well plates of our data set. As correct block size is essential, we tested different block size ranging from 20 to 80. However, after plotting profiles of all data points on a 2D multidimensional scaling (MDS) plot using this platform, we found that the distances between compounds in the plot (Figure S6a) did not reflect the similarities or dissimilarities in the images observed by eye. For example, the negative control (DMSO) data points are closely located to data points of the positive control Arq 197 with concentration 3.16 µM in the MDS plot even though these two conditions showed clearly discrete phenotypes (Figure S6b,c).

CellProfiler is segmentation dependent software. It is able to calculate morphological parameters including Zernike moments, object intensity parameters, topological parameters, texture parameters and image intensity parameters. In total we measured 395 parameters (including the per-image mean and standard deviation for object measurement) for each well using CellProfiler. After robust *z*-score normalization, we applied PCA and plotted the concentration trajectories for the active compounds. However, these trajectories were not biological activity specific (Figure S7a) which was also reflected in the hierarchical clustering result which did not show co-clustering of compounds with the same biological target (Figure S7b). However, we did not try to obtain subpopulation features with this method, which may have improved the clustering results. Finally we applied Mahalanobis distance (α = 0.05) to identify the active concentrations of the biologically active compounds, and those concentrations were used for classification. The lowest error rate of 26.8% was obtained when SVC with linear kernel was applied with 14 parameters selected. Compared to the classification error rate of 12.8% obtained using our method, this higher error rate indicates that subpopulation information which is lacking in CellProfiler plays a very important role in our method and should be taken into account for compound characterization

### Reproducibility of our methodology

To validate the reproducibility of our multi-parametric profiling platform, the 4T1 cell screen was repeated independently on a different occasion. Ten biologically active compounds were included in this screen and we obtained similar results as in the first screen (see Figure S8).

### Other Applications: classification of breast cancer cell lines

**Figure 5:**
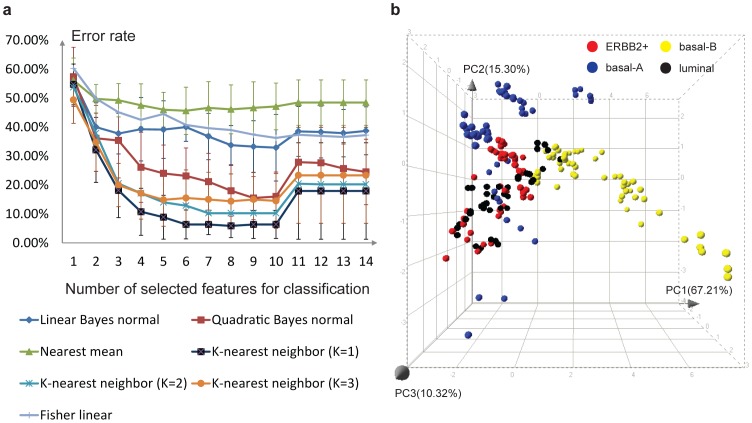
Classification of human breast cancer cell lines. (a) According to the cross-validation result, the smallest error rate was achieved when 8 features were selected. (b) A 3 dimensional PCA plot was generated based on these 8 selected features. Percentages of data variation preserved in each principle component were shown with each axis. Different categories of breast cancer cells are colored differently to show the separation between the various human breast cancer cell classes.

To investigate the wider applicability of our multi-parametric image analysis platform, we used it to classify 44 known human breast cancer cell lines (Table S5) that have been categorized as basal-A, basal-B, luminal or luminal/ERBB2+ based on their gene expression profiles [28]–[31]. The cell lines were cultured in ECM-rich hydrogel in 384-well high content imaging micro plates, with each cell line having 3 or 6 replicates. Obtaining of image stacks and data analysis were as described for the experiments with the 4T1 cells. After robust z-score normalization, we applied supervised forward feature selection with criterion Mahalanobis distance and different classification methods for categorizing basal-A, basal-B, luminal and ERBB2+ cell lines. 10-fold cross-validation was used to assess classification performance (Figure 5a). The mean classification error was lowest when 8 features were selected and 1-nearest neighbor classification method was used, resulting in an error rate of 5.9%. The selected features include intensity parameters from the whole population and subpopulations, morphological parameters and topological parameters from the whole population and subpopulations. Based on these 8 selected features, a PCA was applied (Figure 5b), which clearly shows the separation between the various human breast cancer cell classes. Excluding parameters from the subpopulations resulted in a significantly increased classification error rate (24.9%). Similarly, omitting wavelets, moment and intensity features from our feature set increased the error rate to 35%.

## Discussion

In this study, we developed a new methodology for 3D cell culturing in conjunction with high-throughput imaging and image analysis for the characterization of compounds according to their effect on the phenotype of cultured micro-tissues. Our method is the first for high-content analysis of 3D micro-tissues that is capable of classifying complex phenotypes of 3D cell cultures and can do this in an automated, high-throughput fashion. Our method requires only an initial human-based step (defining branched and spheroidal structures) to train an automatic classifier. This simple procedure is then followed up by a largely automated method to classify multi-cellular structures and retrieve the subpopulation related parameters. The classifier can be applied for a broad class of phenotypes. For example, we applied it to successfully classify 44 different breast cancer cell lines (this manuscript) and also to invasive prostate cancer cells and invasive lung cancer cells (not yet published). If we sub-classify a very different kind of biology, such as tubular versus non-tubular kidney epithelium or villous versus non-villous colon epithelium (other examples of applications we have tried), we would redefine the sub-classes.

This platform can be used for many different applications including identification of biologically active compounds, and concentration dependent trajectory construction. We also demonstrated that our platform can be used to correctly classify a collection of human breast cancer cell lines into known subclasses. Although the group of Bissell et al. have pioneered automated phenotyping of human breast cancer cell lines [32], a complete classification of these cell lines could only be achieved so far with more elaborate and expensive techniques such as gene expression analysis. Recently, a promising new method for nuclear segmentation of 3D cell cultures using curvature-based partitioning was presented [33]. This was used to compare the morphogenesis of four breast cancer cell lines in terms of colony organization. However, this method uses confocal microspcopy images and it is not known whether it would be possible to classify breast cancer cell lines with this method.

For each application of our present methodology, the selected features for phenotype characterization included intensity parameters, morphological parameters and topological parameters from the whole population and subpopulations Taken together, these findings indicate that to systematically study phenotypes associated with modulation of different cellular pathways, it is necessary to quantify images with a full spectrum of phenotypic information. It will not only reveal details that are otherwise not resolved, but also enable adaption to different cell lines or new biological questions. Although the use of 598 parameters may seem redundant initially, it enables us to analyze 3D-cellular phenotypes under a wide variety of conditions, while our feature selection methodologies automatically identify those features that contribute most to the separation and characterization of the particular phenotypes under study.

Compared to other methods, we demonstrated that our methodology offers significant advantages in terms of recognition of specific phenotypes within still reasonable computational demands (see File S1). While some other methods such as PhenoRipper [26] are suitable for analyzing sub-cellular data in 2D imaging-based assays, they are not appropriate for the type of images used in this study. Another advantage of our methodology is that it can be incorporated in the user friendly, freely available ImageJ environment written in Java, and therefore can be run with various operating systems (Linux, Windows, Mac OS X).

## Supporting Information

Figure S1 **The experiment layout of a 384-well micro plate.** Compounds are indexed from C1 to C9 on this plate. DMSO is control. The different shades of grey represents the 6 different concentrations used, increasing from 0.03 µM to 10 µM. The first and last two rows and columns remained empty to avoid an edge effect.
(TIF)

Figure S2 **Subpopulation classification.** (a) Segmentation results of a projected Rhodamine stained f-actin image. (b) Manually selected spherical objects and (c) branched objects. (d) Skeleton of each binary object. (e) Features calculated from each binary object for subpopulation classification. (f) Cross-validation result for comparing different classification methods and identifying optimal number of features for classification. Average error rate of a 10-fold cross-validation is shown in the chart with standard deviation as error bars.
(TIF)

Figure S3 **Mouse breast cancer cell (4T1) exposed to different compounds in 3D cell culture.** For a clear representation of cellular phenotypic responses to different compounds, these images were acquired by a Nikon Eclipse Ti microscope in confocal mode. We used a dry air lens with 4X magnification and 0.2 NA. Two channels (Hoechst stained nuclei channel and Rhoadamine stained F-actin channel) z-stack of 32 xy epifluorescence image slices were collected from each well, with acquisition step size in z direction 50 µm. Maximum intensity projection was applied to compress 3D image stacks to 2D image representation. Concentration of all compounds shown here was 0.316 µM. Scale bar represents 100 micrometer. (a) Untreated cells cultured in 0.2% DMSO, (b) cells exposed to compound Arq 197, (c) cells exposed to dasatinib, (d) cells exposed to entinostat, (e) cells exposed to sorafenib tosylate, (f) cells exposed to bortezomib.
(TIF)

Figure S4 **3D PCA plot of all 29 compounds and concentrations.** Compounds are marked with different colors and the concentration is represented by the size of data points. Percentages of data variation preserved in each principle component are shown with each axis.
(TIF)

Figure S5 **Identification of biologically active compounds.** (a) Comparison of three multi-parametric tests for the identification of biologically active compounds. “positive” indicates correctly identified active concentration of a test compound (p value <α). “False positive” indicates the concentration which is identified as active but no obvious difference was observed compared to control images. “negative” indicates correctly identified inactive concentration of a test compound (p value > =  α). “False negative” indicates the concentration which is identified as inactive but obvious differences were observed compared to control images. “#” means “number of”. (b) Natural logarithm of Mahalanobis distance to DMSO control of all active compounds. Compounds are marked with different colors and shapes. Black dashed line corresponds to the distance with p-value  = 0.05.
(TIF)

Figure S6 **Comparison to PhenoRipper.** (a) A two dimensional MDS plot after using PhenoRipper for the analysis of the compound screen in 4T1 cells. The block size used was 50. Compounds are identified by their color. The highlighted orange point corresponds to a well treated with Arq 197 at a concentration of 3.16 µM, and the highlighted purple point corresponds to a control well. (b–c) Phenotype images corresponding to the highlighted points in (a).
(TIF)

Figure S7
**Comparison to CellProfiler.** (a) A 2D PCA plot of phenotype profiles for negative control (DMSO) and 13 active compounds at different concentrations using CellProfiler to profile compounds in the 4T1 screen (not all compounds are shown). Percentages of data variation preserved in each principle component are shown with each axis. Compounds are marked with different shapes and colors. Compounds with the same biological target are colored the same. Red: BCR-ABL target inhibitor; Yellow: VEFGR inhibitor; Green: EGFR inhibitor; Purple: HDAC inhibitor; Blue: c-MET inhibitor. Concentration is represented by the size of data points. The trend lines were added for each effective compound using polynomial regression model with order two. (b) Hierarchical clustering result for all active compounds using an average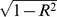 matrix as distance matrix. The scale of dendrogram is the natural logarithm of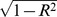.(TIF)

Figure S8 **Reproducibility of our methodology.** (a) A two dimensional PCA plot of phenotype profiles for negative control (DMSO) and 11 active compounds from a repeated experiment. Percentages of data variation preserved in each principle component are shown with each axis. Compounds are marked with different shapes and colors. Compounds with the same biological target are colored the same. Red: BCR-ABL target inhibitor; Yellow: VEFGR inhibitor; Green: EGFR inhibitor; Purple: HDAC inhibitor; Blue: c-MET inhibitor. Concentration is represented by the size of data points. The trend lines were added for each effective compound using polynomial regression model with order two. (b) Classification result using multiple classification methods. Feature selection and classification algorithms are the same as in the first experiment.
(TIF)

Table S1 **Compounds used in the 4T1 cell 3D micro-tissue screen.**
(DOC)

Table S2 **Morphological, moment and intensity parameters measured for the whole object population, raw intensity images, and subpopulations.**
(DOC)

Table S3 **Top 5 parameters which contribute most to each of the 3 first principle components in the 4T1 cell screen.**
(DOC)

Table S4 **Biologically active compounds and corresponding active concentration identified using Mahalanobis distance (α = 0.05).**
(DOC)

Table S5 **Breast cancer cell lines (basal-A, basal-B, luminal or ERBB2+) used for classification.**
(DOC)

File S1 **Image analysis for multi-parametric phenotype profiling, identification of the biologically active compounds, and phenotypic trajectory modeling using 2nd order polynomial regression model.**
(DOC)
