# Supplementary figures and images for: Ultra High Content Image Analysis and Phenotype Profiling of 3D Cultured Micro-Tissues
Source: PLoS One. 2014 Oct 7;9(10):e109688. doi: 10.1371/journal.pone.0109688 (PMC4188701; doi:10.1371/journal.pone.0109688)

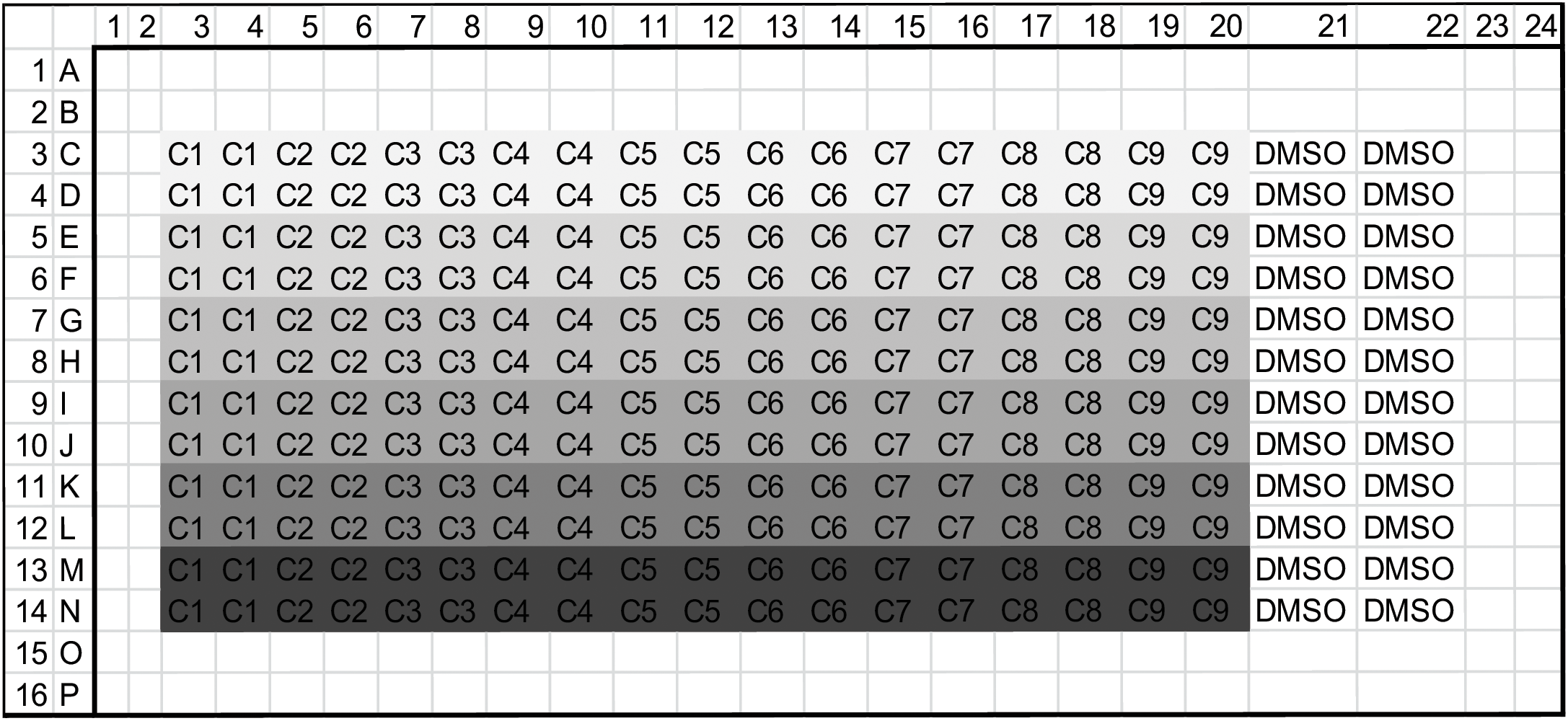

Supplement: Figure S1 — The experiment layout of a 384-well micro plate. Compounds are indexed from C1 to C9 on this plate. DMSO is control. The different shades of grey represents the 6 different concentrations used, increasing from 0.03 µM to 10 µM. The first and last two rows and columns remained empty to avoid an edge effect. (TIF) [file pone.0109688.s001.tif]

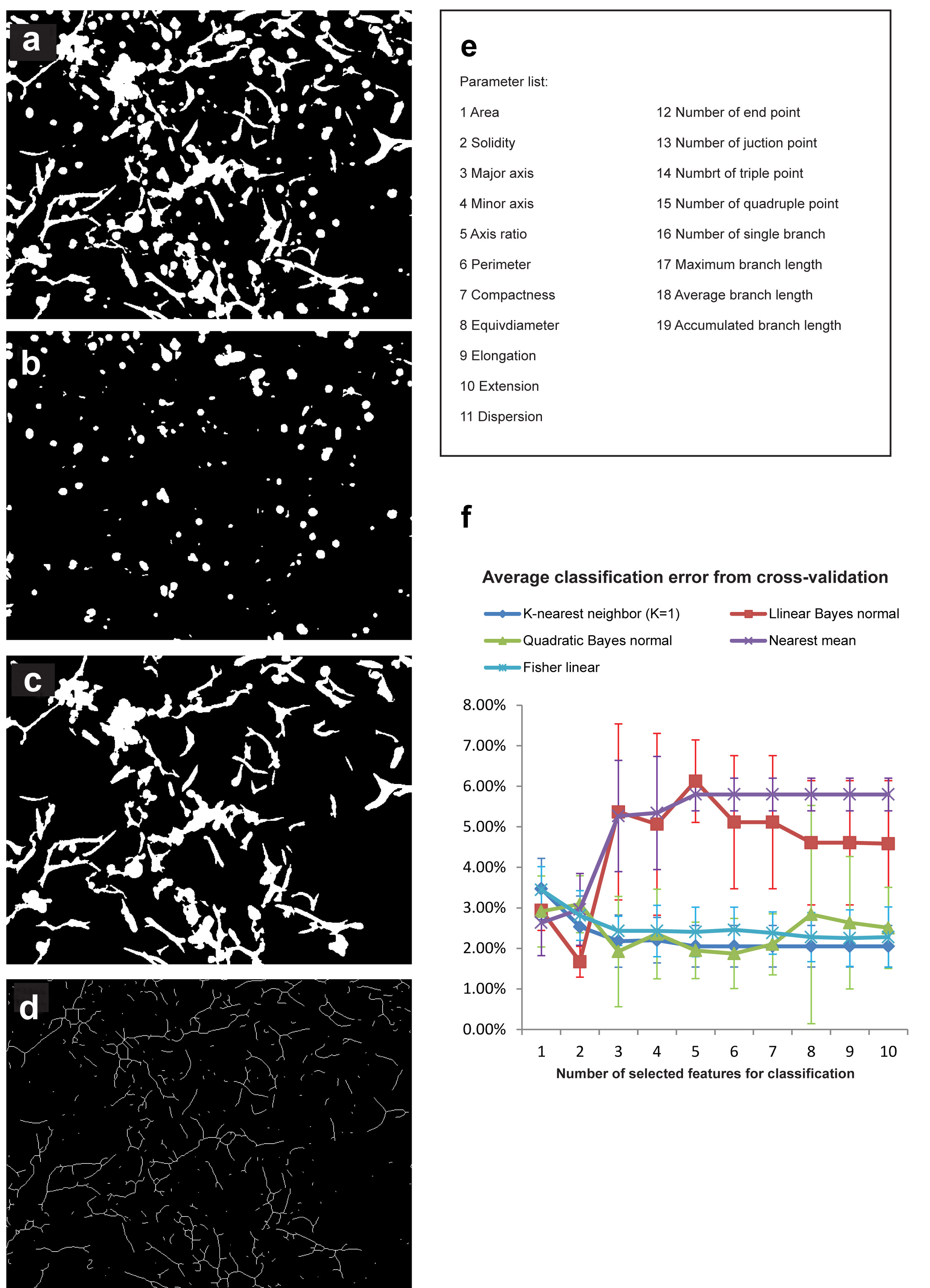

Supplement: Figure S2 — Subpopulation classification. (a) Segmentation results of a projected Rhodamine stained f-actin image. (b) Manually selected spherical objects and (c) branched objects. (d) Skeleton of each binary object. (e) Features calculated from each binary object for subpopulation classification. (f) Cross-validation result for comparing different classification methods and identifying optimal number of features for classification. Average error rate of a 10-fold cross-validation is shown in the chart with standard deviation as error bars. (TIF) [file pone.0109688.s002.tif]

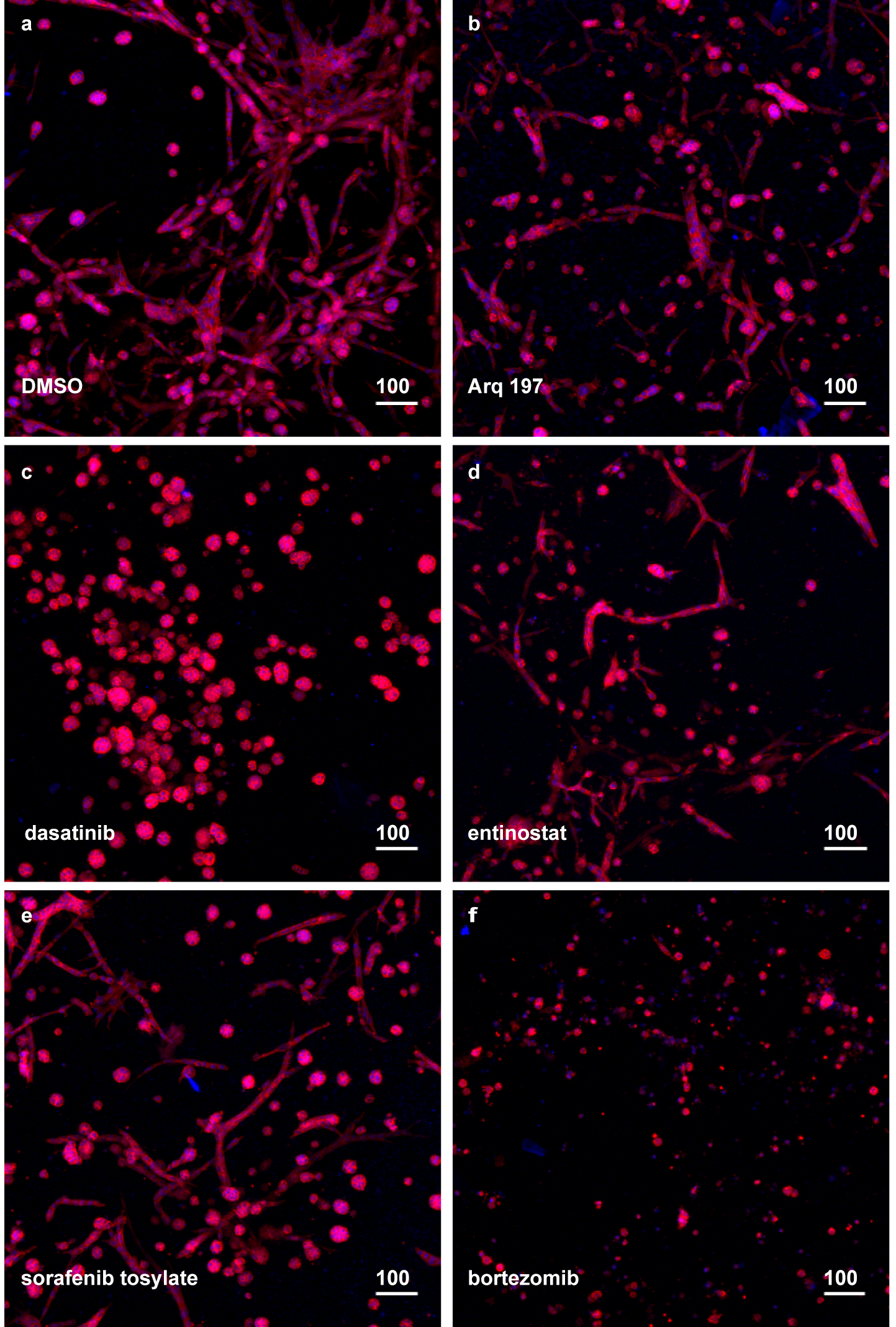

Supplement: Figure S3 — Mouse breast cancer cell (4T1) exposed to different compounds in 3D cell culture. For a clear representation of cellular phenotypic responses to different compounds, these images were acquired by a Nikon Eclipse Ti microscope in confocal mode. We used a dry air lens with 4X magnification and 0.2 NA. Two channels (Hoechst stained nuclei channel and Rhoadamine stained F-actin channel) z-stack of 32 xy epifluorescence image slices were collected from each well, with acquisition step size in z direction 50 µm. Maximum intensity projection was applied to compress 3D image stacks to 2D image representation. Concentration of all compounds shown here was 0.316 µM. Scale bar represents 100 micrometer. (a) Untreated cells cultured in 0.2% DMSO, (b) cells exposed to compound Arq 197, (c) cells exposed to dasatinib, (d) cells exposed to entinostat, (e) cells exposed to sorafenib tosylate, (f) cells exposed to bortezomib. (TIF) [file pone.0109688.s003.tif]

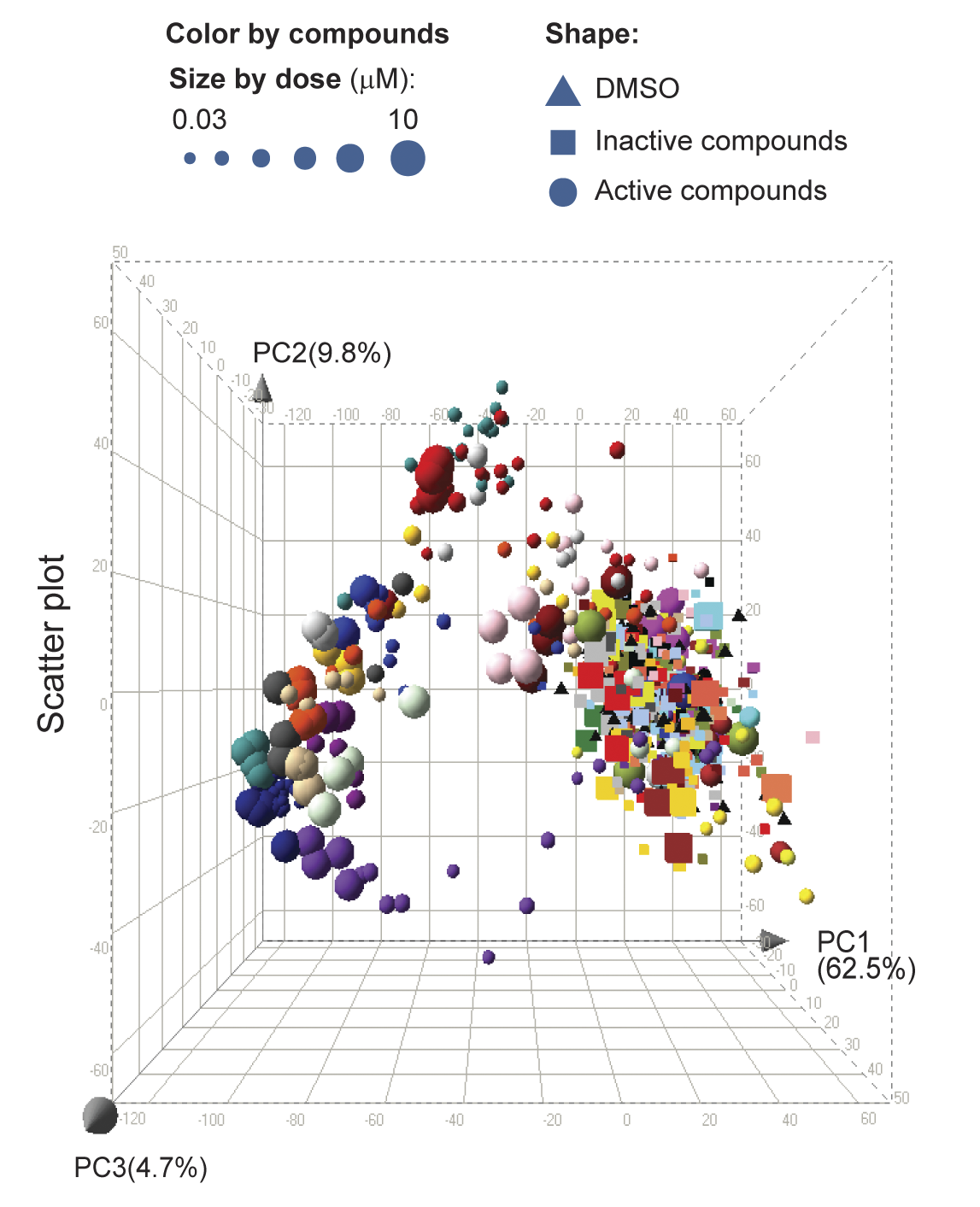

Supplement: Figure S4 — 3D PCA plot of all 29 compounds and concentrations. Compounds are marked with different colors and the concentration is represented by the size of data points. Percentages of data variation preserved in each principle component are shown with each axis. (TIF) [file pone.0109688.s004.tif]

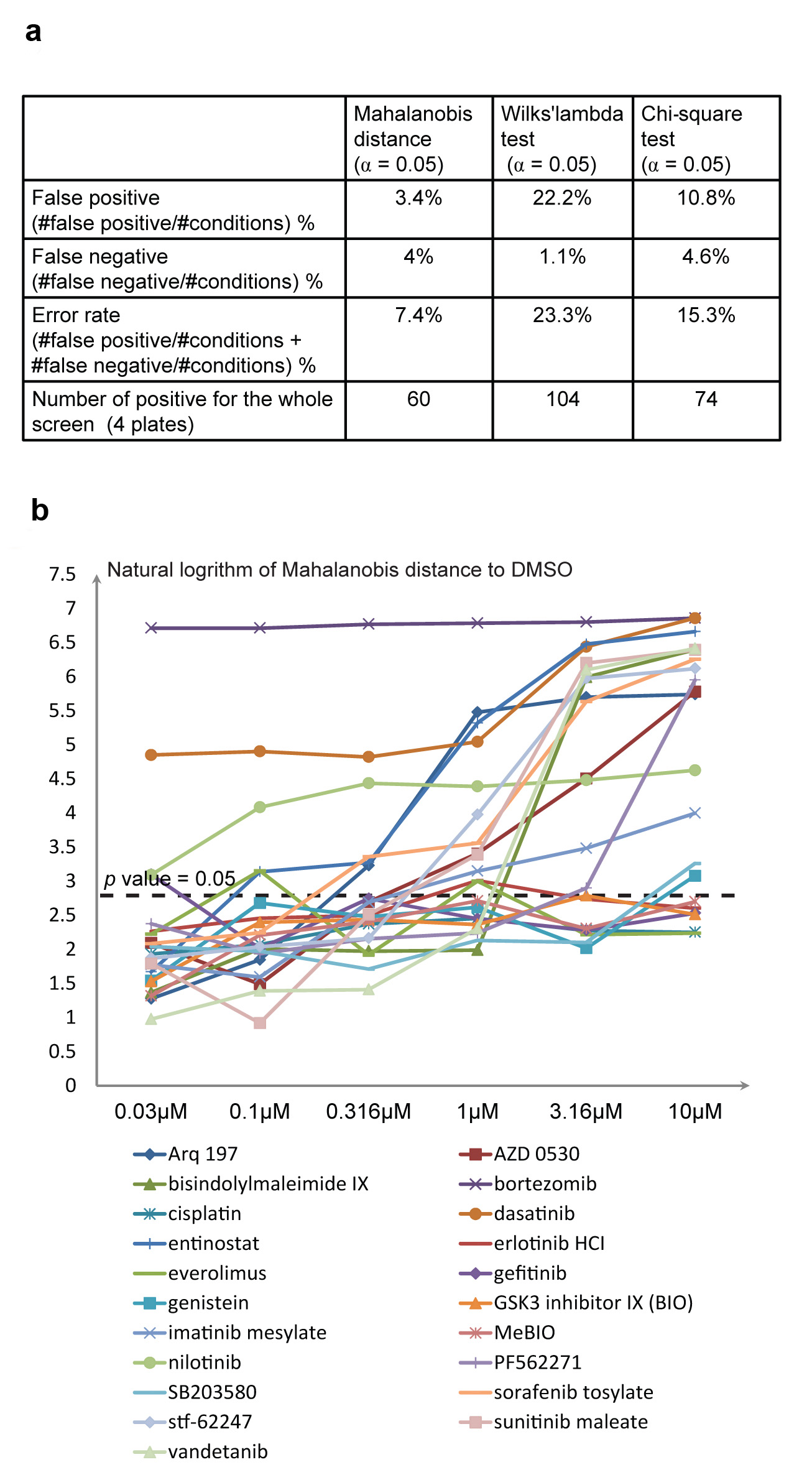

Supplement: Figure S5 — Identification of biologically active compounds. (a) Comparison of three multi-parametric tests for the identification of biologically active compounds. “positive” indicates correctly identified active concentration of a test compound (p value <α). “False positive” indicates the concentration which is identified as active but no obvious difference was observed compared to control images. “negative” indicates correctly identified inactive concentration of a test compound (p value > = α). “False negative” indicates the concentration which is identified as inactive but obvious differences were observed compared to control images. “#” means “number of”. (b) Natural logarithm of Mahalanobis distance to DMSO control of all active compounds. Compounds are marked with different colors and shapes. Black dashed line corresponds to the distance with p-value = 0.05. (TIF) [file pone.0109688.s005.tif]

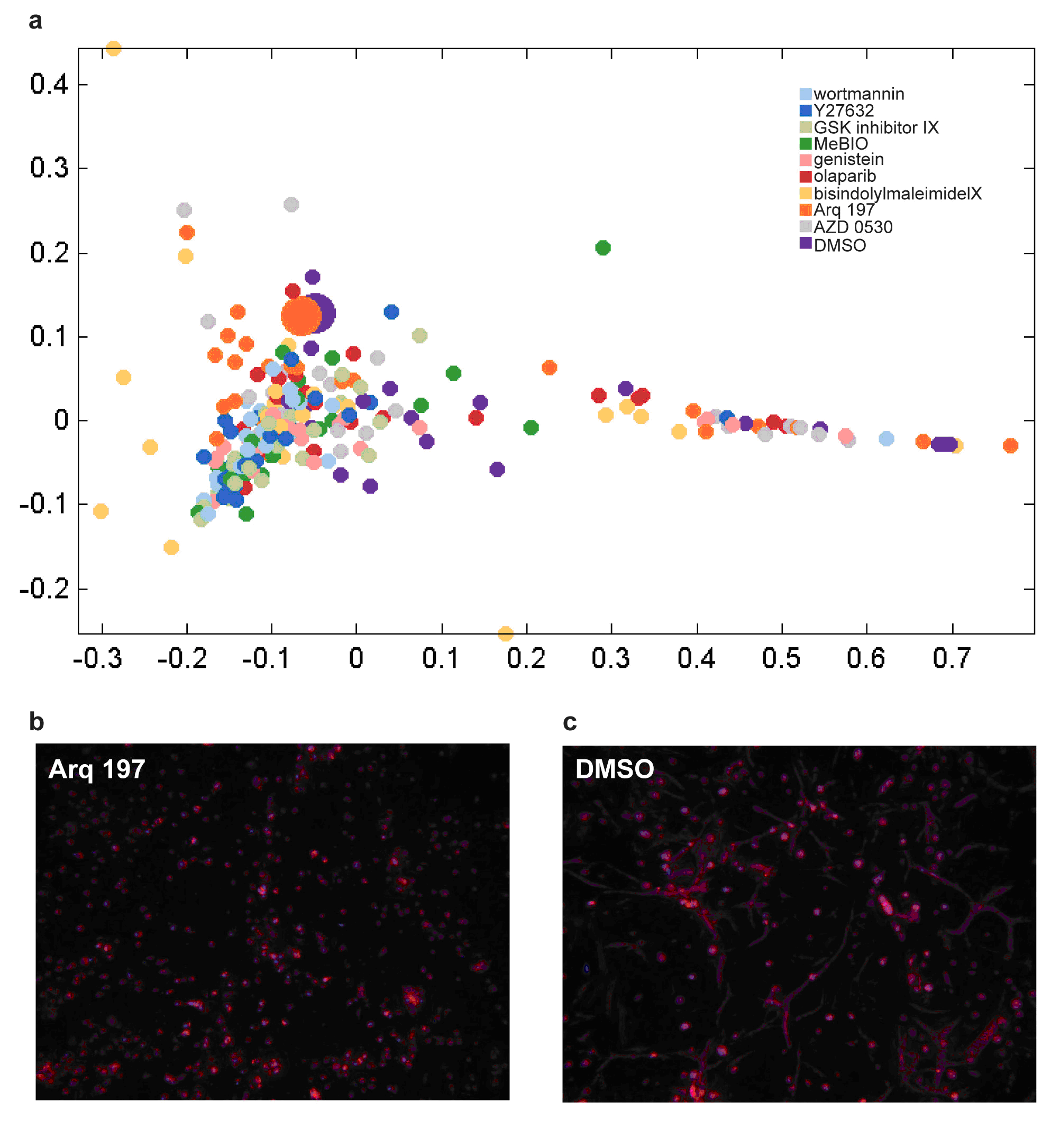

Supplement: Figure S6 — Comparison to PhenoRipper. (a) A two dimensional MDS plot after using PhenoRipper for the analysis of the compound screen in 4T1 cells. The block size used was 50. Compounds are identified by their color. The highlighted orange point corresponds to a well treated with Arq 197 at a concentration of 3.16 µM, and the highlighted purple point corresponds to a control well. (b–c) Phenotype images corresponding to the highlighted points in (a). (TIF) [file pone.0109688.s006.tif]

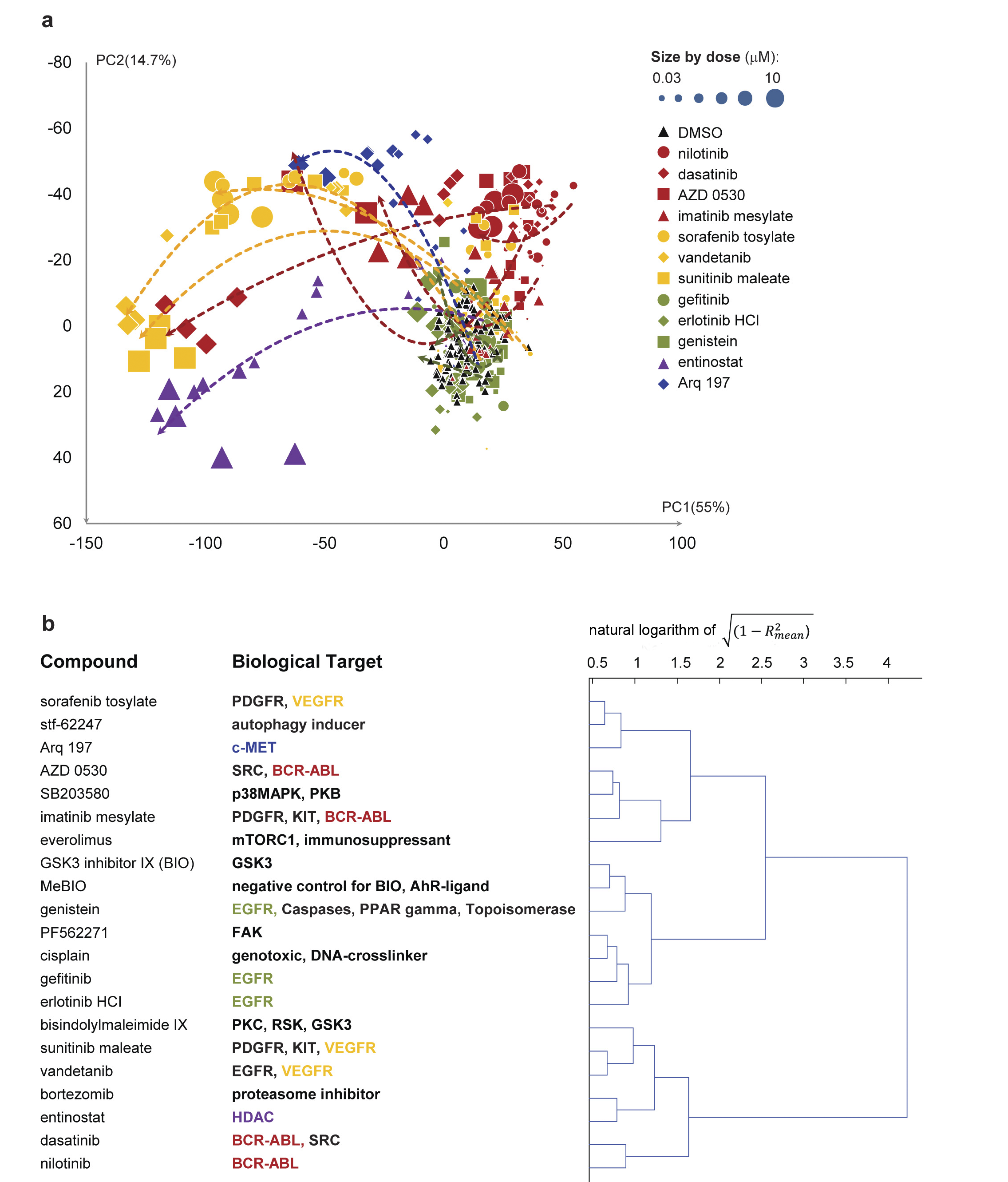

Supplement: Figure S7 — Comparison to CellProfiler. (a) A 2D PCA plot of phenotype profiles for negative control (DMSO) and 13 active compounds at different concentrations using CellProfiler to profile compounds in the 4T1 screen (not all compounds are shown). Percentages of data variation preserved in each principle component are shown with each axis. Compounds are marked with different shapes and colors. Compounds with the same biological target are colored the same. Red: BCR-ABL target inhibitor; Yellow: VEFGR inhibitor; Green: EGFR inhibitor; Purple: HDAC inhibitor; Blue: c-MET inhibitor. Concentration is represented by the size of data points. The trend lines were added for each effective compound using polynomial regression model with order two. (b) Hierarchical clustering result for all active compounds using an average matrix as distance matrix. The scale of dendrogram is the natural logarithm of. (TIF) [file pone.0109688.s007.tif]

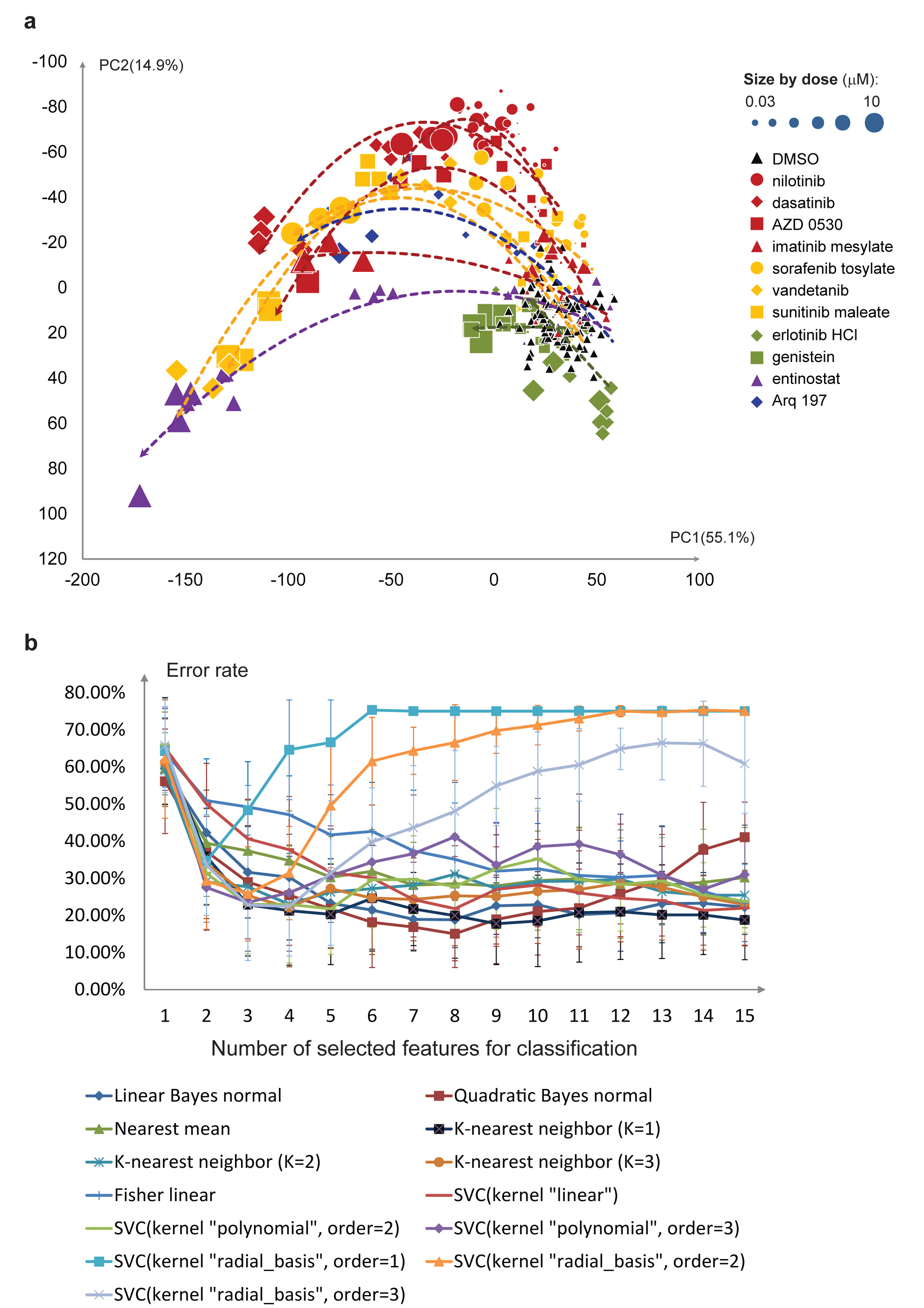

Supplement: Figure S8 — Reproducibility of our methodology. (a) A two dimensional PCA plot of phenotype profiles for negative control (DMSO) and 11 active compounds from a repeated experiment. Percentages of data variation preserved in each principle component are shown with each axis. Compounds are marked with different shapes and colors. Compounds with the same biological target are colored the same. Red: BCR-ABL target inhibitor; Yellow: VEFGR inhibitor; Green: EGFR inhibitor; Purple: HDAC inhibitor; Blue: c-MET inhibitor. Concentration is represented by the size of data points. The trend lines were added for each effective compound using polynomial regression model with order two. (b) Classification result using multiple classification methods. Feature selection and classification algorithms are the same as in the first experiment. (TIF) [file pone.0109688.s008.tif]
