# Supplementary material for: Ultra High Content Image Analysis and Phenotype Profiling of 3D Cultured Micro-Tissues
Source: PLoS One. 2014 Oct 7;9(10):e109688. doi: 10.1371/journal.pone.0109688 (PMC4188701; doi:10.1371/journal.pone.0109688)
Supplement: Table S1 — Compounds used in the 4T1 cell 3D micro-tissue screen. (DOC) [file pone.0109688.s009.doc]

***Supporting Table S1. Compounds used in the 4T1 cell 3D micro-tissue screen***

| **Active in screen** | **Biological activity** |
| --- | --- |
| Arq 197  AZD 0530  bisindolylmaleimide IX  bortezomib  cisplatin  dasatinib  entinostat  erlotinib HCl  everolimus  gefitinib  genistein  GSK3 inhibitor IX (BIO)  imatinib mesylate  MeBIO  nilotinib  PF562271  SB203580  sorafenib tosylate  stf-62247  sunitinib maleate  vandetanib | tyrosine kinase inhibitor (c-MET), positive control  tyrosine kinase inhibitor (SRC family, BCR-ABL)  serine/threonine kinase inhibitor (PKC, RSK, GSK3)  proteasome inhibitor  genotoxic, DNA-crosslinker  tyrosine kinase inhibitor (SRC family, BCR-ABL)  HDAC inhibitor  tyrosine kinase inhibitor (EGFR)  threonine kinase inhibitor (mTORC1), immunosuppressor  tyrosine kinase inhibitor (EGFR)  tyrosine kinase (EGFR), Caspase, PPARγ, and Topo II inhibitor  serine/threonine kinase inhibitor (GSK3)  tyrosine kinase inhibitor (PDGFR, KIT, BCR-ABL)  negative control for BIO, AhR-ligand  tyrosine kinase inhibitor (BCR-ABL)  tyrosine kinase inhibitor (FAK, PTK2B)  serine/threonine kinase inhibitor (p38MAPK, PKB)  tyrosine kinase inhibitor (VEGFR, PDGFR)  autophagy inducer  tyrosine kinase inhibitor (PDGFR, KIT, VEGFR)  tyrosine kinase inhibitor (EGFR, VEGFR) |
| **Inactive in screen** |  |
| DMSO  AG 538  lapatinib dihydrate  LY364947  olaparib  SP600125  SU6668  wortmannin  Y27632 | negative control  tyrosine kinase inhibitor (EGFR)  tyrosine kinase inhibitor (EGFR, HER2)  serine/threonine. kinase inhibitor (TGF-β, RI/RII, p38 MAPK)  PARP inhibitor  serine/threonine. kinase inhibitor (JNK1/2/3, Aurora A, Flt3)  tyrosine kinase inhibitor ( PDGFR,FGFR1, Flk-1/KDR)  phosphoinositide (PI) 3-kinase inhibitor  serine/threonine kinase inhibitor (ROCK) |
