# Supplementary material for: Ultra High Content Image Analysis and Phenotype Profiling of 3D Cultured Micro-Tissues
Source: PLoS One. 2014 Oct 7;9(10):e109688. doi: 10.1371/journal.pone.0109688 (PMC4188701; doi:10.1371/journal.pone.0109688)
Supplement: Table S2 — Morphological, moment and intensity parameters measured for the whole object population, raw intensity images, and subpopulations. (DOC) [file pone.0109688.s010.doc]

| ***Supporting Table S2. Morphological, moment and intensity parameters measured for the whole object population, raw intensity images, and subpopulations*** | |
| --- | --- |
|  |  |
| **List of classic morphological parameters calculated for the whole object population** | |
| Parameter index | Description |
| 1 | #nuclei |
| 2 | #cell colonies |
| 3 | Total area of nuclei |
| 4 | Total area of cell colonies |
| 5 | Mean density of nuclei |
| 6 | Mean density of cell colonies |
| 7 | Average area of cell colonies |
| 8 | Standard deviation of cellular area |
| 9 | Average solidity of cell colonies |
| 10 | Standard deviation of cellular solidity |
| 11 | Average major axis of cell colonies |
| 12 | Standard deviation of cellular major axis |
| 13 | Average minor axis of cell colonies |
| 14 | Standard deviation of cellular minor axis |
| 15 | Average axis ratio of cell colonies |
| 16 | Standard deviation of cellular axis ratio |
| 17 | Average perimeter of cell colonies |
| 18 | Standard deviation of cellular perimeter |
| 19 | Average compactness of cell colonies |
| 20 | Standard deviation of cellular compactness |
| 21 | Average equivdiameter of cell colonies |
| 22 | Standard deviation of cellular equivdiameter |
| 23 | Average feret’s diameter of cell colonies |
| 24 | Standard deviation of cellular feret’s diameter |
| 25 | Average minFeret of cell colonies |
| 26 | Standard deviation of cellular minFeret |
| 27 | Average elongation of cell colonies |
| 28 | Standard deviation of cellular elongation |
| 29 | Average extension of cell colonies |
| 30 | Standard deviation of cellular extension |
| 31 | Average dispersion of cell colonies |
| 32 | Standard deviation of cellular dispersion |
| 33 | Average #end point of cell colonies |
| 34 | Standard deviation of cellular #end point |
| 35 | Average #junction point of cell colonies |
| 36 | Standard deviation of cellular #junction point |
| 37 | Average #single junction point of cell colonies |
| 38 | Standard deviation of cellular #single junction point |
| 39 | Average #single branch of cell colonies |
| 40 | Standard deviation of cellular #single branch |
| 41 | Average #triple point of cell colonies |
| 42 | Standard deviation of cellular #triple point |
| 43 | Average #quadruple point of cell colonies |
| 44 | Standard deviation of cellular #quadruple point |
| 45 | Average maximum branch length of cell colonies |
| 46 | Standard deviation of cellular maximum branch length |
| 47 | Average branch length of cell colonies |
| 48 | Standard deviation of cellular branch length |
| 49 | Average branch length (only between 2 single junction points) of cell colonies |
| 50 | Standard deviation of cellular branch length (only between 2 single junction points) |
| 51 | Average accumulated branch length of cell colonies |
| 52 | Standard deviation of cellular accumulated branch length |
| 53 | Maximum branch length of cell colonies |
| 54 | Accumulated branch length of cell colonies |
|  | (#) means “number of” |
|  |  |
| **List of moment parameters calculated for the whole object population** | |
| Parameter index | Description |
| From 1-49 | The mean of the first 49 Zernike moments measured on the binary mask of the cellular colonies |
| From 50-98 | The standard deviation of the first 49 Zernike moments measured on the binary mask of the cellular colonies |
|  |  |
| **List of intensity parameters calculated for the raw intensity projected images** | |
| Parameter index | Description |
| 1 | Total intensity of Hoechst channel |
| 2 | Total intensity of Rhodamine channel |
| 3 | Mean intensity of Hoechst channel |
| 4 | Mean intensity of Rhodamine channel |
| 5 | Intensity standard deviation of Hoechst channel |
| 6 | Intensity standard deviation of Rhodamine channel |
| 7 | Maximum intensity of Hoechst channel |
| 8 | Maximum intensity of Rhodamine channel |
| 9 | Minimum intensity of Hoechst channel |
| 10 | Minimum intensity of Rhodamine channel |
| From 11-80 | 70 Gabor wavelet (We used four scale S=4, and six orientation K=6) of Rhodamine channel |
| From 81-129 | 49 Zernike moments of Rhodamine channel |
| From 130-136 | 7 Hu moments of Rhodamine channel |
|  |  |
| **List of morphological and intensity parameters calculated for the subpopulations** | |
| Parameter index | Description |
| 1 | #cell colonies which are classified as spherical object |
| 2 | #cell colonies which are classified as branched object |
| 3 | Accumulated area of cell colonies which are classified as spherical object |
| 4 | Accumulated area of cell colonies which are classified as branched object |
| 5 | Accumulated branch length of cell colonies which are classified as spherical object |
| 6 | Accumulated branch length of cell colonies which are classified as branched object |
| From 7-52 | The classic morphological parameters(parameter index from 7 to 52) calculated from the cell colonies which are classified as spherical object |
| 53 | Mean intensity of cell colonies which are classified as spherical object |
| 54 | Intensity standard deviation of cell colonies which are classified as spherical object |
| 55 | Mean(intensity standard deviation of each cell colony which is classified as spherical object) |
| 56 | Std(intensity standard deviation of each cell colony which is classified as spherical object) |
| 57 | Mean(maximum intensity of each cell colony which is classified as spherical object) |
| 58 | Std(maximum intensity of each cell colony which is classified as spherical object) |
| 59 | Mean(minimum intensity of each cell colony which is classified as spherical object) |
| 60 | Std(minimum intensity of each cell colony which is classified as spherical object) |
| From 61-106 | The classic morphological parameters (parameter index from 7 to 52) calculated from the cell colonies which are classified as branched object |
| 107 | Mean intensity of cell colonies which are classified as branched object |
| 108 | Intensity standard deviation of cell colonies which are classified as branched object |
| 109 | Mean(intensity standard deviation of each cell colony which is classified as branched object) |
| 110 | Std(intensity standard deviation of each cell colony which is classified as branched object) |
| 111 | Mean(maximum intensity of each cell colony which is classified as branched object) |
| 112 | Std(maximum intensity of each cell colony which is classified as branched object) |
| 113 | Mean(minimum intensity of each cell colony which is classified as branched object) |
| 114 | Std(minimum intensity of each cell colony which is classified as branched object) |
| From 115-212 | The mean and standard deviation of the first 49 Zernike moments of cell colonies which are classified as spherical object |
| From 213-310 | The mean and standard deviation of the first 49 Zernike moments of cell colonies which are classified as branched object |
|  | (#) means “number of” |
|  | (Mean()) indicates the formula to calculate the mean |
|  | (Std())indicates the formula to calculate the standard deviation |
