# Supplementary material for: Ultra High Content Image Analysis and Phenotype Profiling of 3D Cultured Micro-Tissues
Source: PLoS One. 2014 Oct 7;9(10):e109688. doi: 10.1371/journal.pone.0109688 (PMC4188701; doi:10.1371/journal.pone.0109688)
Supplement: Table S3 — Top 5 parameters which contribute most to each of the 3 first principle components in the 4T1 cell screen. (DOC) [file pone.0109688.s011.doc]

***Supporting Table S3. Top 5 parameters which contribute most to each of the 3 first principle components in the 4T1 cell screen***

| **Principle component 1 (62.45%)*** | **Principle component 2 (9.81%)** | **Principle component 3 (4.69%)** |
| --- | --- | --- |
| -Average dispersion of cell colonies which are classified as branched object  -Average extension of cell colonies which are classified as branched object  -Average solidity of cell colonies which are classified as branched object  -Average Feret’s diameter of cell colonies  Standard deviation of cellular solidity | -Average solidity of cell colonies which are classified as branched object  -Mean intensity of cell colonies which are classified as branched object  -Mean(minimum intensity of each cell colony which is classified as branched object)  -Mean(maximum intensity of each cell colony which is classified as branched object)  -Intensity standard deviation of Rhodamine channel | -Average solidity of cell colonies which are classified as branched object  -Average extension of cell colonies which are classified as branched object  -Average elongation of cell colonies which are classified as branched object  -Average Zernike moments (order 4) measured on the binary mask of the cellular colonies  -Average Zernike moments (order 14) measured on the binary mask of cellular colonies which are classified as branched object |
