# Supplementary material for: Ultra High Content Image Analysis and Phenotype Profiling of 3D Cultured Micro-Tissues
Source: PLoS One. 2014 Oct 7;9(10):e109688. doi: 10.1371/journal.pone.0109688 (PMC4188701; doi:10.1371/journal.pone.0109688)
Supplement: Table S4 — Biologically active compounds and corresponding active concentration identified using Mahalanobis distance (α = 0.05). (DOC) [file pone.0109688.s012.doc]

***Supporting Table S4***: Biologically active compounds and corresponding active concentration identified using Mahalanobis distance (α=0.05)

| Active compounds | Active concentrations | | | | | |
| --- | --- | --- | --- | --- | --- | --- |
| Arq 197 | 10 μM | 3.16 μM | 1 μM | 0.316 μM |  |  |
| AZD 0530 | 10 μM | 3.16 μM | 1 μM |  |  |  |
| bisindolylmaleimide IX | 10 μM | 3.16 μM |  |  |  |  |
| bortezomib | 10 μM | 3.16 μM | 1 μM | 0.316 μM | 0.1 μM | 0.03 μM |
| cisplatin |  |  | 1 μM |  |  |  |
| dasatinib | 10 μM | 3.16 μM | 1 μM | 0.316 μM | 0.1 μM | 0.03 μM |
| entinostat | 10 μM | 3.16 μM | 1 μM | 0.316 μM | 0.1 μM |  |
| erlotinib HCl |  | 3.16 μM | 1 μM |  |  |  |
| everolimus |  |  | 1 μM |  | 0.1 μM |  |
| gefitinib |  |  |  | 0.316 μM |  | 0.03 μM |
| genistein | 10 μM |  |  |  |  |  |
| GSK3 inhibitor IX |  | 3.16 μM |  |  |  |  |
| imatinib mesylate | 10 μM | 3.16 μM | 1 μM |  |  |  |
| MeBIO (neg. control for BIO) | 10 μM |  |  |  |  |  |
| nilotinib | 10 μM | 3.16 μM | 1 μM | 0.316 μM | 0.1 μM | 0.03 μM |
| PF562271 | 10 μM | 3.16 μM |  |  |  |  |
| SB203580 | 10 μM |  |  |  |  |  |
| sorafenib tosylate | 10 μM | 3.16 μM | 1 μM | 0.316 μM |  |  |
| stf-62247 | 10 μM | 3.16 μM | 1 μM |  |  |  |
| sunitinib maleate | 10 μM | 3.16 μM | 1 μM |  |  |  |
| vandetanib | 10 μM | 3.16 μM |  |  |  |  |
