# Supplementary material for: Ultra High Content Image Analysis and Phenotype Profiling of 3D Cultured Micro-Tissues
Source: PLoS One. 2014 Oct 7;9(10):e109688. doi: 10.1371/journal.pone.0109688 (PMC4188701; doi:10.1371/journal.pone.0109688)
Supplement: Table S5 — Breast cancer cell lines (basal-A, basal-B, luminal or ERBB2+) used for classification. (DOC) [file pone.0109688.s013.doc]

***Supporting Table S5: Breast cancer cell lines (basal-A, basal-B, luminal or ERBB2+) used for classification***

| Cell line index | Cell line | Categories | Replicates |
| --- | --- | --- | --- |
| 1 | BT20 | basal-A | 6 |
| 2 | BT474 | ERBB2+ | 3 |
| 3 | BT483 | luminal | 3 |
| 4 | BT549 | basal-B | 6 |
| 5 | CAMA-1 | luminal | 6 |
| 6 | EVSA-T | ERBB2+ | 6 |
| 7 | HCC1143 | basal-A | 6 |
| 8 | HCC1395 | basal-B | 6 |
| 9 | HCC1569 | basal-A | 3 |
| 10 | HCC1806 | basal-A | 3 |
| 11 | HCC1937 | basal-A | 3 |
| 12 | HCC1954 | basal-A | 3 |
| 13 | HCC202 | ERBB2+ | 3 |
| 14 | HCC70 | basal-A | 6 |
| 15 | Hs578T | basal-B | 6 |
| 16 | MCF7 | luminal | 6 |
| 17 | MDA-MB-134VI | luminal | 3 |
| 18 | MDA-MB-175VII | luminal | 3 |
| 19 | MDA-MB-231 | basal-B | 6 |
| 20 | MDA-MB-361 | ERBB2+ | 3 |
| 21 | MDA-MB-415 | luminal | 3 |
| 22 | MDA-MB-435s | basal-B | 6 |
| 23 | MDA-MB-436 | basal-B | 6 |
| 24 | MDA-MB-453 | ERBB2+ | 6 |
| 25 | MDA-MB-468 | basal-A | 6 |
| 26 | MPE600 | luminal | 3 |
| 27 | OCUB-F | ERBB2+ | 6 |
| 28 | OCUB-M | ERBB2+ | 6 |
| 29 | SK-BR-3 | ERBB2+ | 6 |
| 30 | SK-BR-7 | basal-B | 6 |
| 31 | SUM102PT | basal-B | 6 |
| 32 | SUM1315MO2 | basal-B | 6 |
| 33 | SUM149PT | basal-A | 6 |
| 34 | SUM159PT | basal-B | 6 |
| 35 | SUM185PE | luminal | 6 |
| 36 | SUM190PT | ERBB2+ | 3 |
| 37 | SUM225CWN | ERBB2+ | 3 |
| 38 | SUM229PE | basal-A | 6 |
| 39 | SUM44PE | luminal | 3 |
| 40 | SUM52PE | luminal | 3 |
| 41 | T47D | luminal | 3 |
| 42 | UACC812 | ERBB2+ | 3 |
| 43 | UACC893 | ERBB2+ | 3 |
| 44 | ZR-75-1 | luminal | 3 |

* The human breast cancer cell lines were from ATCC (Manassas, VA, USA) or as described by Hollestelle et al. (12) and provided to us by Prof. Dr. John A. Foekens and Dr. John W. Martens from Erasmus University Medical Center-Daniel den Hoed Cancer Center, Rotterdam, The Netherlands. Details of the ATCC cell lines can be obtained from: https://www.lgcstandards-atcc.org/Products/Cells_and_Microorganisms/Cell_Lines/Human.aspx
