# Supplementary material for: Ultra High Content Image Analysis and Phenotype Profiling of 3D Cultured Micro-Tissues
Source: PLoS One. 2014 Oct 7;9(10):e109688. doi: 10.1371/journal.pone.0109688 (PMC4188701; doi:10.1371/journal.pone.0109688)
Supplement: File S1 — Image analysis for multi-parametric phenotype profiling, identification of the biologically active compounds, and phenotypic trajectory modeling using 2nd order polynomial regression model. (DOC) [file pone.0109688.s014.doc]

# Ultra high content analysis and phenotype profiling of 3D cultured micro tissues

Zi Di, Maarten J D Klop, Vasiliki-Maria Rogkoti, Sylvia E Le Dévédec, Bob van de Water, Fons J Verbeek, John H N Meerman & Leo S Price

# Supporting File S1

## Image analysis for multi-parametric phenotype profiling

**a. 2D projection**. In order to achieve high-throughput in our methodology, we used wide-field microscopy for screening as its imaging process is much faster than confocal laser scanning microscopy. However, due to its limited depth of field, each image slice obtained from a wide-field microscope includes both in-focus regions and out-of-focus regions of specimen that are bigger than the depth of field. To extract only the in-focus information, a free open source plugin of ImageJ “Stack Focuser” was used to compose 2D image slices by projecting only in-focus regions from each slice of image stacks. Firstly, a median filter with kernel size 3$\times$3 pixels was used to remove the noise. We empirically defined the filter kernel size as 3$\times$3; using a bigger size would affect object size. Next, a Sobel filter (Gonzalez and Woods, 2002) was used for edge detection and subsequently a maximum filter was applied to map the local focal strength, by taking the maximum value in a kernel of the specified size. In this project, we defined the kernel size as

10$\times$10 pixels, which is slightly bigger than the average size of the nuclei (8$\times$8 pixels). In the last step, for each coordination $\left(x,y\right)$ the pixel value from the slice with the highest focal strength was copied to the composed 2D image (Fig. 2c, 2d).

**b. Segmentation**. For the Hoechst stained nuclei channel, watershed masked clustering (WMC) (Cao *et al.,* 2011; Yan and Verbeek, 2012) was applied to retrieve the binary masks for individual nuclear regions. This algorithm first generates watersheds on the Gaussian filter (σ = 2.0) convolved images to separate the adjacent nuclei into individual compartments. Convolving with a Gaussian filter prevented the influence of noise from causing artificial local maxima. Next, K-means clustering was then applied on the images prior to convolving to refine the region of the nucleus in each compartment.

For the Rhodamine stained F-actin channel, median filter (kernel size 3$\times$3 pixels) and rolling ball (radius = 30 pixels) were applied before segmentation to remove the background and reduce the noise level. The radius of rolling ball was chosen to be slightly bigger than the smallest cell colonies. Next, the local Niblack algorithm (Niblack, 1986) was used to define regions of cell colonies. This algorithm calculates a threshold $T\left(x,y\right)$ for each pixel $\left(x,y\right)$ according to the intensity mean $mean\left(x,y\right)$ and standard deviation $\sigma\left(\mathrm{x},\mathrm{y}\right)$ calculated within a specified kernel size (we defined this size as 30 X 30 pixels which is slightly bigger than the smallest cell colonies) centered at pixel $\left(x,y\right)$:

$T\left(x,y\right)=mean\left(x,y\right)+k*\sigma\left(x,y\right)$ [S1]

where $k$ indicates how much the standard deviation influences the threshold and its value was determined empirically.

**c. Subpopulation classification.** To automatically identify the spherical clusters and branched networks in the 3D micro-tissues, a classifier was trained. Five test image stacks were randomly selected from DMSO controls which contain both spherical structures and tubular structures**.** 2D projection and segmentation were applied for the Rhodamine stained F-actin channel to define the binary masks of cellular regions (Supporting Fig. S2a). In total 1318 binary cellular masks were obtained. Next, we manually identified spherical objects (Supporting Fig. S2b) and branched objects (Supporting Fig. S2c). For each of binary masked objects, a skeleton (Supporting Fig.S2d) was retrieved using a “Skeletonize” plugin of ImageJ, which iteratively removes pixels from the edges of objects in a binary image, reducing them to single pixel wide skeletons. We labeled each pixel of the skeleton with one of the following categories depending on their neighbors in 8 connectivity:

1: End point: the pixel with only 1 skeleton pixel in neighbor.

2: Junction point: the pixel with more than 2 skeleton pixels in neighbor.

3: Single junction point: the connected junction points in 8 connectivity.

4: Slab point: the pixel with 2 skeleton pixels in neighbor

5: Triple point: the pixel with 3 skeleton pixels in neighbor.

6: Quadruple point: the pixel with 4 skeleton pixels in neighbor.

Nineteen parameters were finally calculated from each binary object, including basic morphological parameters and topological parameters from the skeletons (Supporting Fig. S2e). The topological parameters were calculated based on the category of each skeleton pixel defined above. Single branch is defined as the part of skeleton between single junction points, end points, or single junction point and end point. Branch length of a single branch is defined as the sum of Euclidian distance between two adjacent skeleton pixels over all pixels on the single branch. The definition and equation of the 11 morphological parameters are defined as follows:

1: Area: number of pixel in the binary object.

2: Solidity: $\frac{Area}{Areaofconvexhull}$

3: Major axis: the primary axis of the best fitting ellipse derived from 2^nd^ order moment $\eta$ (Gonzalez and Woods, 2002)

4: Minor axis: the secondary axis of the best fitting ellipse derived from 2^nd^ order moment $\eta$(Gonzalez and Woods, 2002)

5: Axis ratio: $\frac{MajorAxis}{MinorAxis}$

6: Perimeter: the length of the contour of the binary object.

7: Compactness: $\frac{Perimeter^{2}}{\text{4π}*Area}$

8: Equivdiameter: the length of the diameter of the perfect circle that had the same area as the binary object.

9: Elongation: measures how much the shape must be compressed along its major axis in order to minimize the extension (Gonzalez and Woods, 2002)

10: Extension: measures how much the shape differs from the circle (Gonzalez and Woods, 2002)

11: Dispersion: minimum extension that can be attained by uniform compression of the shape (Gonzalez and Woods, 2002), where

$\eta=\frac{\mu_{pq}}{\mu_{pq}^{\gamma}}$, $\gamma=\frac{p+q}{2}+1$,$\mu_{pq}=\sum_{0}^{width-1} \sum_{0}^{height-1} \left(x-\overline{x}\right)^{p}\left(y-\overline{y}\right)^{q}B\left(x,y\right)$

$width$ and $height$ indicate the width and height of images. $\left(\overline{x},\overline{y}\right)$ is the center coordinate of the binary object $B\left(x,y\right).$

For classification, we used a Matlab toolbox, PRTools. Firstly, feature selection was performed to avoid the curse of dimensionality, using the search algorithm ‘forward’ with criterion “Mahalanobis distance” (Mahalanobis, 1936). For this two subpopulations situation, Mahalanobis distance criterion optimizes:

$J_{maha}=\left(\mu_{2}-\mu_{1}\right)^{T}\left(\frac{\Sigma_{1}+\Sigma_{2}}{2}\right)^{-1}\left(\mu_{2}-\mu_{1}\right)$ [S2]

where $\mu_{1},\mu_{2},\Sigma_{1},\Sigma_{2}$ indicate the mean vector of subpopulation 1 data, mean vector of subpopulation 2 data, the covariance matrices of subpopulation 1 and 2. To define the optimal number of features, multiple classification methods were applied and the 10-fold cross-validation was applied to evaluate the number of features, as well as the accuracy of each classification methods. The classification methods tested were k-nearest neighbor classification (k=1), linear Bayes normal classification (Duda *et al.*, 2001; Liu and Wechsler, 2000; Webb, 2002), quadratic Bayes normal classification (Duda *et al.*, 2001; Webb, 2002) nearest mean classification, and Fisher linear classification (Duda *et al.*, 2001; Webb, 2002; Raudys and Duin, 1998).

Supporting Fig. S2f shows the average error rate obtained from 10-fold cross-validation for each classification method calculated for a certain number of features. The classification error rate was calculated according to equation [1] in the manuscript. In this study, prior probability was defined equally for each of two subpopulation classes. The result revealed that when 2 features were selected, linear Bayes normal classification gave the least average error rate of 1.67%. The 2 features which were selected most often are perimeter and major axis. Finally, a linear Bayes normal classifier based on these 2 parameters was trained and embedded in the platform to automatically classify all cellular binary masks of the whole experiment.

**d. Quantification.** Quantification algorithms were assembled from the literature (Bakal, *et al.*, 2007; Wang *et al.*, 2008) and incorporated into an ImageJ plugin to extract phenotypic parameters from the images. Those parameters can be categorized into three groups according to their function: classical morphological parameters, moment parameters and intensity-based parameters. Classical morphological parameters include a series of shape properties that were calculated on the binary masks or on the skeletons which were obtained from the binary masks. Moment parameters were either calculated on the binary masks as shape descriptors, or on the projected image to retrieve intensity distribution information. In this study, we only considered the moments which are invariant under translation and rotation, including Zernike moments up to degree 12 (Wang *et al.*, 2008; Abdel Qader *et al.*, 2007) and Hu moments (Hu, 1962). The intensity-based parameters were calculated on the projected image, including basic intensity properties such as the average intensity of the image, and texture features like Gabor wavelets (Hu, 1962; Manjunath and Ma, 1996). For each of the classic morphological parameters and moment parameters which were measured on the binary objects, the mean and standard deviation were calculated over the whole population, as well as each subpopulation class (Supporting Table S1), providing 598 parameters to profile the phenotype of each well.

## Identification of the biologically active compounds

**a. Normalization.** A simple statistics robust z-score (Birmingham, *et al.*, 2009) was calculated for each of 598 parameters and used for plate normalization and further data analysis:

$robustz-scoreforeachparameter=\frac{x_{i}-median_{negative}}{MAD_{negative}}$ [S3]

where $x_{i}$ is the original measurement on the $i^{'th}$ well, $median_{negative}$and $MAD_{negative}$are the median and median absolute deviation of this parameter calculated for the negative control. Compared with z-score, robust z-score is less sensitive to outliers.

**b. Principle component analysis.** PCA was performed to project the normalized 598 z-scores to independent principle component space. We obtained 9 principle components which retained 90% of data variation. We plotted the data points (all compounds with different concentrations) on the first 3 dimensional PC space and 6 outliers were observed. These outliers were confirmed by visual inspection of the original image stacks and were due to errors occurring at the image acquisition stage. Since the outliers would result in bias in the PCA, they were removed and PCA was performed again on the outlier removed dataset. As the result, another 9 principle components were obtained which maintained 90% of dataset variation.

**c. Multi-parametric test.** For hits identification, we compared three commonly used multivariate tests: Mahalanobis distance (Mahalanobis, 1936; McLachlan, 1992), Chi-square test (Greenwood and Nikulin, 1996) and Wilks’ lambda (Mardia *et al*., 1979). They were performed on the 9 principle components obtained from the PCA. The Mahalanobis distance $D$ calculates the similarity of a data vector to the negative control based on the correlation between variables:

$D_{i}^{2}=\left(x_{i}-\mu_{negative}\right)^{T}\Sigma_{negative}^{-1}\left(x_{i}-\mu_{negative}\right)$ [S4]

where $x_{i}$ is a 9 principle components vector of the *i*’th well and $\mu_{negative}$ indicates mean vector of the negative control. $\Sigma_{negative}$ indicates the covariance matrix of the negative control. Mahalanobis distance of each replicate was calculated and the average was calculated over replicates to assess significant level.

Chi-square calculates

$\chi^{2}=\left(x_{i}-\mu_{negative}\right)^{2}$ [S5]

When the variables are normal distributed, both $D_{i}^{2}$ and $\chi^{2}$ should follow a Chi-square distribution with $n$ degrees of freedom ($n$ is number of variables). Wilks' lambda is a statistics used as a measure of the class center separation when classes are multinomial with identical covariance matrix:

$\Lambda=\frac{det\left(S_{wg}\right)}{det\left(S_{wg}+S_{bg}\right)}$ [S6]

where $det$indicates determinant of a matrix, $S_{wg}$ and $S_{bg}$ represent within-group and between-group sum-of-square and cross-products matrix (SSCP) correspondingly, when two groups in comparison are defined as negative control and tested compound with certain concentration. For all three methods, *p*-value was calculated and appropriate threshold of significance level α was calibrated by manually checking a selection of control and compound-treated images.

## Phenotypic trajectory modeling using 2nd order polynomial regression model

To further characterize the different phenotypes induced by biologically active compounds, we used 2^nd^ order polynomial regression model to build the phenotypic concentration trajectory for each identified active compound. For each compound we used all concentrations including inactive concentrations, active concentrations and the average of DMSO controls for the regression model. We firstly calculated the data variation for each of the 9 principle components. Two sample Kolmogorov-Smirnov test was used to compare control data and active compounds data. The principle components with no significant difference between control and compounds, equal data variation, or bigger variation in negative controls were excluded to avoid over training of the regression model. As the result, the first 2 components were retained. Next, a 2D polynomial regression model of the trajectory for each compound was trained. To quantify the distance between two trajectories, for example the trajectories of compound $i$ and compound$j$, we firstly calculated the $R_{(i,j)}^{2}$using the trajectory model built for compound $i$ to fit the data points of compound$j$, and also for the other way around $R_{(j,i)}^{2}$.

$R_{(i,j)}^{2}=1-\frac{SS_{err(i,j)}}{SS_{(i,j)}}$ [S7]

$SS_{err(i,j)}=\sum_{n_{j}} (x_{2}-x'_{2})^{2}$ [S8]

$SS_{tot(i,j)}=\sum_{n_{j}} (x_{2}-\overline{x_{2}})^{2}$ [S9]

where $x_{2}$ is the observed value of a data point of compound $j$on the PC2. $x'_{2}$ is the predicted value of the same data point of compound $j$ on the PC2 using the trajectory model of compound .$\overline{x_{2}}$is the mean of $x_{2}$over all data points of compound$j$. $n_{j}$ represents number of data points for compound $j$. Finally the difference between the trajectories of two compounds $i,j$ was computed based on the coefficient of determination $R^{2}$:

$Distance\left(i,j\right)=\sqrt{1-R_{mean}^{2}}$ [S10]

$R_{mean}^{2}=\frac{R_{\left(i,j\right)}^{2}+R_{\left(j,i\right)}^{2}}{2}$ [S11]

where $R_{\left(i,j\right)}^{2}$ is the coefficient of determination to indicate how well data points of compound $j$ fit a trajectory which is modeled for compound $i$. Subsequently, a hierarchical clustering with complete linkage (Defays, 1977) was applied on the distance matrix defined above (Supporting Fig. S4a).

This modeling method can be easily extended to more dimensions. For example, to model a 3D trajectory, our task becomes to fit a model

$$\begin{matrix} x'_{2}=\beta_{a}x_{1}^{2}+\beta_{b}x_{1}+\varepsilon_{1} \\ x'_{3}=\beta_{c}x_{1}^{2}+\beta_{d}x_{1}x'_{2}+\beta_{e}x_{1}+\beta_{f}x'_{2}^{2}+\beta_{g}x'_{2}+\varepsilon_{2} \end{matrix}$$

for each compound, where $\beta$ represents regression coefficient and $\varepsilon$ is an error term. $x_{3}$ and $x'_{3}$ is the observed value and the predicted value of a data point of compound $j$on the 3^rd^ dimensional principle component, respectively. Then $SS_{err(i,j)}$and $SS_{tot(i,j)}$ would be defined as

$$SS_{err(i,j)}=\sum_{n_{j}} (x_{3}-x'_{3})^{2}$$

$$SS_{tot(i,j)}=\sum_{n_{j}} (x_{3}-\overline{x_{3}})^{2}$$

### *Computational efficiency*

Our complete method takes approximately 260 minutes of computational time for 384 wells on an Intel i7 3770 model with 4 GB of RAM with Windows XP professional 2002. As the costs of these computational systems are relatively modest, we do not regard the computation time of our method as a major drawback because the load of the analysis can easily be spread across multiple computers.

## Software

For image analysis, ImageJ plugins were developed in-house, and written in Java. KNIME was used to developed the hits identification pipeline including robust z-score normalization, PCA and multi-parametric test. Mahalanobis distance, Chi-square and Wilks’ lambda test were coded in R snippets and integrated in KNIME. For phenotypic pattern recognition including clustering, classification and feature selection, we used the Matlab toolbox, PRTool.

Software and all image data are available at:

http://dx.doi.org/10.4121/uuid:d5b91e46-07e7-4077-bd63-3fa2b82c847f

***References***

Abdel Qader,H. *et al.* (2007) Fingerprint recognition using Zernike moments. *Int. Arab J. Inf. Techn.,* **4**, 372-376.

Bakal.C. *et al.* (2007) Quantitative morphological signatures define local signaling networks regulating cell morphology. *Science,* **31,** 1753-1756.

Birmingham,A. *et al.* (2009) Statistical methods for analysis of high-throughput RNA interference screens. *Nat. Methods,* **6**, 569-575.

Cao,L. et al (2011) Pattern recognition in high-content cytomics screens for target discovery: case studies in endocytosis. In Loog,M., Reinders,M.J.T., Ridder,D.D. and Wessels.L. (eds.), *PRIB'11 Proceedings of the 6th IAPR international conference on Pattern recognition in bioinformatics*, Springer-Verlag, Berlin-Heidelberg, pp 330-342.

Defays,D. (1977) Efficient Algorithm for a Complete Link Method. *Comput. J*., **20**, 364-366.

Duda,R.O., *et al.* (2001) *Pattern classification*, 2nd edn. Wiley, New York, p 654.

Gonzalez,R.C. and Woods,R.E. (2002) *Digital image processing,* 2nd edn. Prentice Hall, Upper Saddle River, N.J., pp 136-137.

Greenwood,P.E. and Nikulin,M.S. (1996) *A guide to chi-squared testing.* John Wiley & Son, New York, pp 1-280.

Hu,M. (1962) Visual-Pattern Recognition by Moment Invariants. *IRE Trans. Inform. Theory,*  **8**, 179-187.

Liu,C. and Wechsler,H. (2000) Robust coding schemes for indexing and retrieval from large face databases. *IEEE Trans. Image Process*., **9**, 132-137.

Mahalanobis,P.C, (1936) On the generalised distance in statistics. *Proceedings of the National Institute of Sciences of India,* **2**, 49-55.

Manjunath,B.S. and Ma,W.Y. (1996) Texture features for browsing and retrieval of image data. *Ieee Trans. Pattern Anal.,* **18**, 837-842.

Mardia,K.V. *et al*. (1979) *Multivariate analysis.* Academic Press, London - New York, pp 1-521.

McLachlan,G.J. (1992) *Discriminant analysis and statistical pattern recognition.* John Wiley & Son, New York, pp 52-54.

Niblack,W. (1986) *An introduction to digital image processing*, Prentice-Hall International, Englewood Cliffs, N.J., pp 115-116.

Raudys.S. and Duin,R.P.W. (1998) Expected classification error of the Fisher linear classifier with pseudo-inverse covariance matrix. *Pattern Recogn. Lett*., **19**, 385-392.

Wang,J. *et al*. (2008) Cellular phenotype recognition for high-content RNA interference genome-wide screening. *J. Biomol. Screen*., **13**, 29-39.

Webb,A.R. (2002) *Statistical pattern recognition*, 2nd edn. John Wiley & Sons, Chichester, UK, pp 123-163.

Yan,K. and Verbeek,F.J. (2012) Segmentation for high-throughput image analysis: watershed masked clustering. In Margaria,T. and Steffen,B (eds.) *Proceedings of ISoLA 2012, the 5th International Conference on Leveraging Applications of Formal Methods, Verification and Validation*., Springer-Verlag, Berlin-Heidelberg, pp 25-41.
